# Low temperature conditioning of garlic (*Allium sativum L*.) “seed” cloves induces alterations in sprouts proteome

**DOI:** 10.3389/fpls.2015.00332

**Published:** 2015-05-13

**Authors:** Miguel D. Dufoo-Hurtado, José Á. Huerta-Ocampo, Alberto Barrera-Pacheco, Ana P. Barba de la Rosa, Edmundo M. Mercado-Silva

**Affiliations:** ^1^Laboratorio de Fisiología y Bioquímica Poscosecha de Frutas y Hortalizas, Departamento de Investigación y Posgrado, Facultad de Química, Universidad Autónoma de QuerétaroQuerétaro, Mexico; ^2^Laboratorio de Proteómica y Biomedicina Molecular, División de Biología Molecular, Instituto Potosino de Investigación Científica y Tecnológica A.C.San Luis Potosí, Mexico

**Keywords:** *Allium sativum*, sprouts, cold conditioning, two-dimensional electrophoresis, LC-ESI-MS/MS

## Abstract

Low-temperature conditioning of garlic “seed” cloves substitutes the initial climatic requirements of the crop and accelerates the cycle. We have reported that “seed” bulbs from “Coreano” variety conditioned at 5°C for 5 weeks reduces growth and plant weight as well as the crop yields and increases the synthesis of phenolic compounds and anthocyanins. Therefore, this treatment suggests a cold stress. Plant acclimation to stress is associated with deep changes in proteome composition. Since proteins are directly involved in plant stress response, proteomics studies can significantly contribute to unravel the possible relationships between protein abundance and plant stress acclimation. The aim of this work was to study the changes in the protein profiles of garlic “seed” cloves subjected to conditioning at low-temperature using proteomics approach. Two sets of garlic bulbs were used, one set was stored at room temperature (23°C), and the other was conditioned at low temperature (5°C) for 5 weeks. Total soluble proteins were extracted from sprouts of cloves and separated by two-dimensional gel electrophoresis. Protein spots showing statistically significant changes in abundance were analyzed by LC-ESI-MS/MS and identified by database search analysis using the Mascot search engine. The results revealed that low-temperature conditioning of garlic “seed” cloves causes alterations in the accumulation of proteins involved in different physiological processes such as cellular growth, antioxidative/oxidative state, macromolecules transport, protein folding and transcription regulation process. The metabolic pathways affected include protein biosynthesis and quality control system, photosynthesis, photorespiration, energy production, and carbohydrate and nucleotide metabolism. These processes can work cooperatively to establish a new cellular homeostasis that might be related with the physiological and biochemical changes observed in previous studies.

## Introduction

Garlic (*Allium sativum*) is one of the most economically important *Allium* species, and has been widely cultivated for more than 5000 years. Garlic bulbs have been used as condiments, spices, seasonings, or flavoring as well as for its medicinal value, while garlic leaves are consumed as green vegetables (Ade-Ademilua et al., 2009). The garlic bulb consists of a disc-shaped stem that supports the cloves and it is surrounded by the dried basal sheaths of the foliage leaves. Cloves are considered organs of propagation of the specie and contain a set of leaves, including the storage leaf and the vegetative bud that contains a predominant sprout leaf and several primordia leaves that surround the apical meristem, which activates their growth under certain environmental conditions to generate a new plant.

Growth period and bulb production differ greatly from year to year, planting date and location, because there is a strong genotype-environment interaction. Cold storage, growth temperature, and photoperiod are the main environmental factors affecting the ontogeny in this crop, but the response to these factors depend on phenological stage (Takagi, 1990). The emergence of the sprout is controlled mainly by temperature, in absent of dormancy, whereas bulb initiation is promoted by exposure of cloves to low temperature (environmental or cold conditioning), growth temperature and photoperiod.

Kamenetsky et al. (2004) also showed that the temperature and photoperiod, strongly affect garlic morphology and plant development as well as the leaf elongation, clove formation, and dormancy induction, indicating that the environmental regulatory effect is obligatory and yet quantitative in certain varieties. However, some varieties may show different cold requirements, and the same variety can show differences in different growing areas affecting the commercial production of this crop. Which indicates that the length of storage period as well as the temperature used will have different effects on the responses of garlic plant. In addition to their immediate effects, environmental factors also have long-term effect in each of the development stages.

Maintaining the bulbs before planting at temperatures from 0 to 10°C for a period of 2 months accelerates the cycle and substitutes the initial climatic requirements of the crop. The exposure of garlic “seed” bulbs to low temperatures modifies the hormonal balance, which leads to an early plant development. “Seed” bulbs without cold storage develop only when they receive suitable light period and temperature conditions for their requirements, whereas cold treated seed bulbs begin to develop more quickly at high temperatures than at low temperatures.

The most striking effect of cold conditioning is the increase in earliness, especially in cultivars with greater low temperature requirement for development. However, there are many contradictions about the effects of cold conditioning on the garlic yield; some researchers have observed increases while other reported no significant differences and yet others have reported depressive effects. We have observed that in “seed” bulbs from “Coreano” variety that were conditioned for 16 days at 5°C, there were no effects on the yield and bulbs quality. However, the conditioning during 5 weeks at 5°C accelerated the crop cycle, decreased plant height and increased the synthesis of phenolic compounds and anthocyanins in the outer scale leaves of the bulbs at harvest time compared to plants from “seed” bulbs stored at room temperature. That indicates a possible stress for low temperature (Dufoo-Hurtado et al., 2013; Guevara-Figueroa et al., 2015). Nevertheless, knowledge about what happens during cold conditioning at functional and biochemical levels in garlic is very limited.

Low temperature is a major environmental constraint associated with many structural, physiological, and biochemical changes within plant cells, as well as with altered gene expression patterns (Kosmala et al., 2009). While genomic and transcript-profiling studies have provided a wealth of information about the process of cold acclimation, there is a growing recognition that the abundance of mRNA transcripts is not always a representative of cognate protein levels and that mechanisms of posttranslational regulation must also play an important role (Renaut et al., 2006). Consequently, proteomics provides a complementary approach between the classical physiological approach and molecular tools, especially for research using non-model plant species, where no genomic sequencing data is available or it is limited. Results from proteomic analysis revealed different proteins associated with the plant response to low-temperature environment by being newly synthesized, accumulating or decreasing. Among other pathways, the differentially expressed proteins are involved in signaling, translation, host-defense mechanisms, carbohydrate metabolism and amino acid metabolism, including both well-documented stress-responsive proteins and some novel cold-responsive proteins (Renaut et al., 2006; Kosová et al., 2011). These results have demonstrated the power of the proteomic approach in studies of plant response to low-temperature conditions.

Identification of the changes in the garlic proteome will contribute to the elucidation of the physiological and plant developmental modifications induced by cold conditioning of garlic “seed” cloves. Therefore, the aim of this work was to apply the proteomics tools (two-dimensional gel electrophoresis coupled with tandem mass spectrometry) to study the changes in the protein profiles of garlic “seed” cloves subjected to low-temperature conditioning.

## Material and methods

### Plant materials and low-temperature conditioning

“Seed” bulbs of garlic (*A. sativum*, L.) cv. “Coreano” were provided by the Garlic Producer Association from Aguascalientes and cultivated at Cosio, Aguascalientes, Mexico, during the crop cycle 2011–2012. One hundred and fifty bulbs of garlic cv. “Coreano” harvested in the 2012-2013 season were stored during 4 months at room temperature. These bulbs were separated into two sets of 75 bulbs; one set was maintained at room temperature (RT) (23°C), and the other was conditioned during 5 weeks at low temperature (5°C), both treatments were under dark conditions. At the end of this period, three sets of 25 bulbs (three biological replicates) were separated and threshed and the cloves of medium size (5–6 g) were selected. The garlic sprouts (leaf primordia) of fifteen selected cloves, of each treatment and replicate were collected and pooled and used for the analysis. Each set was frozen in liquid nitrogen and stored at −70°C until their analysis. From each biological replicate, two technical replicates were carried out and processed as independent samples.

### Protein sample preparation

Sprout proteins were extracted from three independent biological replicates, each sprout pool was milled in a coffee grinder (Braun, Naucalpan, Mexico) to a fine powder. Three grams of powder were mixed with 20 mL of cold acetone. The suspension was centrifuged 10 min at 7650× g and 4°C (Super T21; Sorvall, Kendro Laboratory Products, Newtown, CT, USA.). The pellet was washed with 20 mL of cold acetone and centrifuged at the same conditions. The pellet was suspended in 6 mL of extraction buffer (8M urea, 2% w/v 3-[(3-cholamidopropyl)-dimethylammonio]-1-propanesulfonate, 20 mM dithiothreitol, 2 mM phenylmethylsulfonyl fluoride, 0.002% w/v bromophenol blue). The suspension was mixed in a vortex for 1 min, sonicated during 150 s at 35% of amplitude (GE-505, Ultrasonic Processor, Sonics & Materials, Inc., Newtown, CT, USA); the suspension was centrifuged 20 min at 20,220× g at 4°C. The supernatant was filtered with Miracloth (Calbiochem, Darmstadt, Germany) and centrifuged under the same conditions. The supernatant was subjected to protein clean-up according to manufacturer's instructions (ReadyPrep™ 2-D Cleanup Kit, Bio-Rad, Hercules, CA, USA.). The protein pellet was suspended in rehydration buffer (8 M urea, 2% w/v 3-[(3-cholamidopropyl)-dimethylammonio]-1-propanesulfonate, 20 mM dithiothreitol, 0.002% w/v bromophenol blue), mixed in vortex for 30 s and sonicated for 80 s. The suspension was centrifuged under the previous conditions and the supernatant was recovered. Protein concentration was determined by using protein assay (Bio-Rad) with bovine serum albumin (BSA) used as standard.

### Two-dimensional polyacrylamide gel electrophoresis and image analysis

Sprout soluble proteins (1.5 mg) were suspended in 450 μL of rehydration buffer, containing 0.5% v/v IPG buffer pH 4-7 (Bio-Rad), and were loaded onto 24 cm strips, linear immobilized pH gradient 4–7 (IPG) strips (Bio-Rad). Passive rehydration was carried out at room temperature during 14–16 h. The IEF was conducted at 50 mA per IPG strip and 20 °C in an Ettan IPGphor system (GE Healthcare, Piscataway, NJ, USA). The IEF conditions were: (I) 200 V gradient for 2 h; (II) 400 V gradient for 2 h; (III) 1500 V gradient for 2 h; (IV) 4500 V gradient for 3 h; (V) 8000 V gradient for 3 h, and (VI) holding at 8000 V for 10 h. After isoelectric focusing, the IPG strips were stored at −20°C or immediately equilibrated for 15 min in equilibration buffer (50 mM Tris–HCl pH 8.8, 6 M Urea, 30% v/v glycerol, 2% w/v SDS, 0.002% w/v bromophenol blue, 65 mM dithiothreitol). Strips were transferred to a vertical SDS-polyacrylamide gel. The second dimension was performed on 13% polyacrylamide-SDS gels using an Ettan DALT six electrophoresis unit (GE Healthcare), by SDS electrophoresis buffer (25 mM Tris pH 8.8, 192 mM glycine, and 0.1% w/v SDS) and resolved at 20 mA/gel until the dye (bromophenol blue) reached the bottom of the gels. Two technical replicates per each biological replicates were run for both treatments. After SDS–PAGE, gels were stained with PhastGel Blue R (GE Healthcare) and scanned at 100 μm resolution with a Pharos FX plus Molecular Imager (Bio-Rad). Images were analyzed with Melanie v7.0 software (GeneBio, Geneva, Switzerland). Gel image analysis included spot detection, spot measurement, background subtraction, and spot matching. To correct the variability and to reflect quantitative variation, the spot volumes were normalized as the percentage of the total volume of all spots in the gel. The molecular masses of proteins in gels were determined by co-electrophoresis of molecular weight standards (BenchMark Protein Ladder, Invitrogen. Carlsbad, CA, USA), and the isoelectric point of proteins was determined by migration of protein spots on 24 cm IPG linear gradient strips (pH 4–7). Protein spots were considered as differentially accumulated when their normalized volumes displayed a fold change =1.5 when controls and treatments (RT and 5°C, respectively) were compared. Significant changes were determined using *t*-test (*p* < 0.01).

### In-gel digestion and tandem mass spectrometry analysis (LC-ESI-MS/MS)

Selected protein spots were excised from the gels and distained, reduced with 10 mM dithiothreitol in 25 mM ammonium bicarbonate followed by protein alkylation with 55 mM iodoacetamide. Protein digestion was carried out overnight at 37°C with sequencing grade trypsin (Promega, Madison, WI, USA). Nanoscale LC separation of tryptic peptides was performed with a nanoACQUITY UPLC System (Waters, Milford, MA, USA) and tandem mass spectrometry analysis carried out in a SYNAPT HDMS (Waters) as previously reported (Huerta-Ocampo et al., 2014) with a brief modification: Accurate mass data were collected in an alternating data dependent acquisition mode (DDA). In low energy mode, data were collected at constant collision energy of 3 eV. In elevated-energy mode, the collision energy was ramped from 15 to 45 eV during 5 s of integration.

### Protein identification and classification

MS/MS spectra data sets were used to generate PKL files using Protein Lynx Global Server v2.4 (PLGS, Waters). Proteins were then identified using PKL files and the Mascot search engine v2.3 (Matrix Science, London, UK). Searches were conducted against the *Viridiplantae* subset of the NCBInr protein database (2 391 213 sequences, October 2013) and against the *A. sativum* subset of the NCBInr EST database (21,694 sequences, October 2013). Trypsin was used as the specific protease, and one missed cleavage was allowed. The mass tolerance for precursor and fragment ions was set to 10 ppm and 0.1 Da, respectively. Carbamidomethyl cysteine was set as fixed modification and oxidation of methionine was specified as variable modification. Significant Mascot scores (>39 for the *Viridiplantae* subset of the NCBInr protein database or >33 for *A. sativum* subset of the NCBInr EST database) indicating the identity or extensive homology at *p* < 0.05 and the presence of at least two peptides were considered necessary for reliable identification. Identified proteins were classified into different categories of biological processes in which they were involved according to Gene Ontology (http://www.geneontology.org/).

## Results and discussion

### Identification of low-temperature conditioning responsive proteins in garlic sprouts

**Figure 1:**
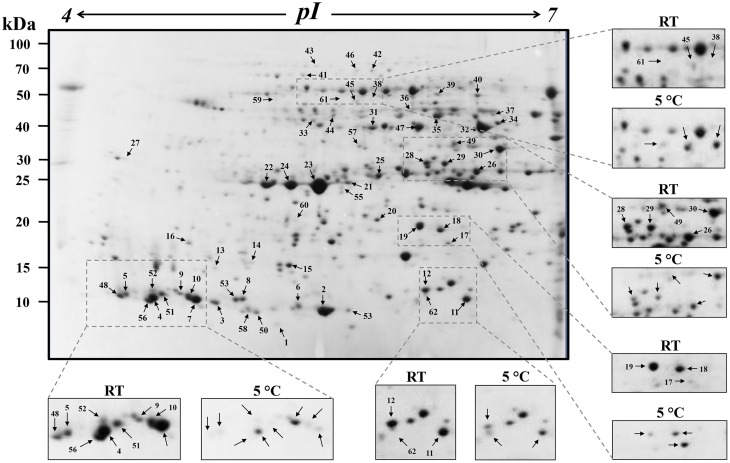
**Representative 2-DE pattern of garlic sprouts proteins from cloves stored at room temperature (RT) or low-temperature conditioning (5°C)**. Spot numbers indicate the protein spots analyzed by tandem mass spectrometry. Zoomed images show the differential accumulation of protein spots between both samples.

Four hundred eighty-five protein spots were reproducibly detected in RT samples and conditioned at 5°C. Sixty two spots showed statistically significant differences at ratios over 1.5-fold in relation with cold conditioning. Figure 1 shows the position of the 62 differentially accumulated protein spots and close-ups of some of the changes in protein spot abundance between treatments. The reproducibility among the technical and biological replicates of the 2-DE gels is shown in magnified images (Supplementary Figures S1–S3)

Among the differentially accumulated protein spots, 22 increased, whereas 37 decreased significantly in abundance in response to low-temperature conditioning. Interestingly three spots appeared only after low-temperature conditioning. All differentially accumulated protein spots were excised from 2-DE gels and subsequently subjected to LC-ESI-MS/MS analysis. Fifty spots (81%) were successfully identified (*p* < 0.05) while in 12 cases (19%) the identification was not possible (spots 1, 2, 28, 29, 30, 40, 48, 50, 52, 56, 58, and 60). In four cases, the same spot matched to more than one protein (Table 1, Supplementary Table S1).

**Table 1:**
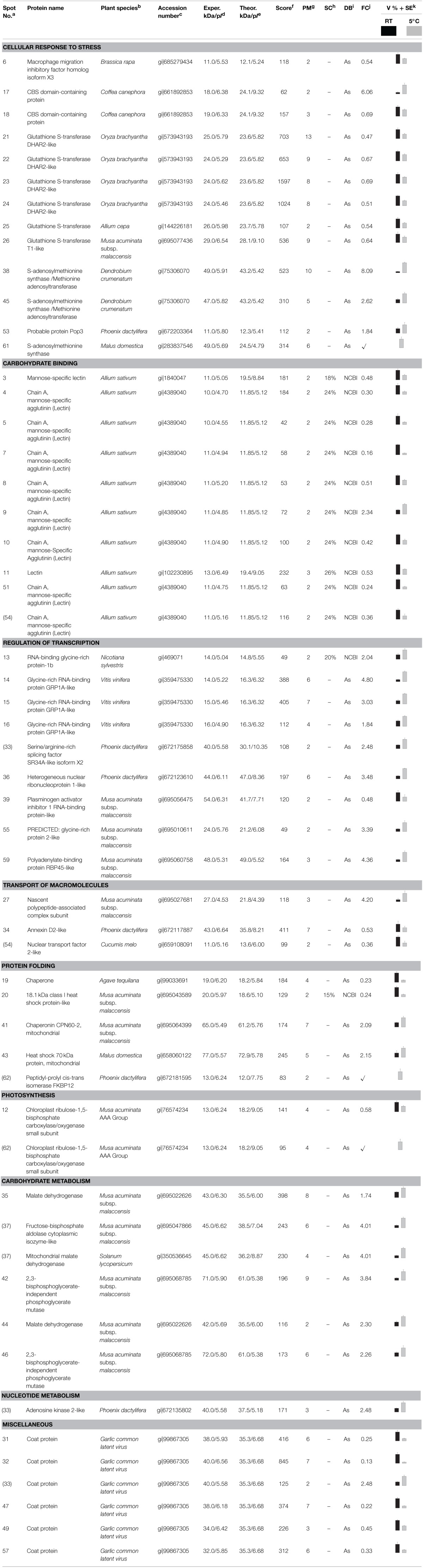
**Identification of differentially accumulated proteins in garlic (*Allium sativum* L.) sprouts subjected to low-temperature conditioning**.

*^a^ Spot numbers as indicated in Figure 1; spot numbers in parentheses indicate that the same spot matched to distinct proteins. ^b^ Plant species represent the most likely orthologous organisms reported in the NCBInr protein database. ^c^ Accession numbers according to NCBInr databse. When the best matches were against the Allium sativum subset of the NCBInr EST database, the most likely orthologous obtained after BLASTX against the NCBInr protein database are reported. ^d^ Experimental mass (kDa) and pI of identified protein spots. ^e^ Theoretical mass (kDa) and pI of identified proteins retrieved from NCBInr database or after calculation using the compute pI/Mw tool (http://web.expasy.org/compute_pi/). ^f^ Mascot score reported after database search. Individual ion scores >23 (for the Allium sativum subset of the NCBInr EST database) or >39 (for the Viridiplantae subset of the NCBInr protein database) are statistically significant (p < 0.05). ^g^ Number of peptides matched. ^h^ Sequence coverage. ^i^ Database. As, Allium sativum subset of the NCBInr EST database; NCBI, Viridiplantae subset of the NCBInr protein database. ^j^ Fold change is expressed as the ratio of the volume % between low-temperature/room-temperature conditioned sprouts, and each value represents the mean value of three biologically independent replicates analyzed by duplicate. For some spots, fold change cannot be accurately calculated because of a complete absence of the spot in room temeprature conditioned samples; this is noted by the symbols ✓, indicating the presence of the spot only in low-temparature conditioned samples. ^k^ Protein spot accumulation changes in Allium sativum sprouts proteins conditioned at room (23°C) or low-temperature (5°C) for 5 weeks. Each column represents the mean value of three biologically independent replicates analyzed by duplicate. Error bars indicate ± standard error (SE)*.

**Figure 2:**
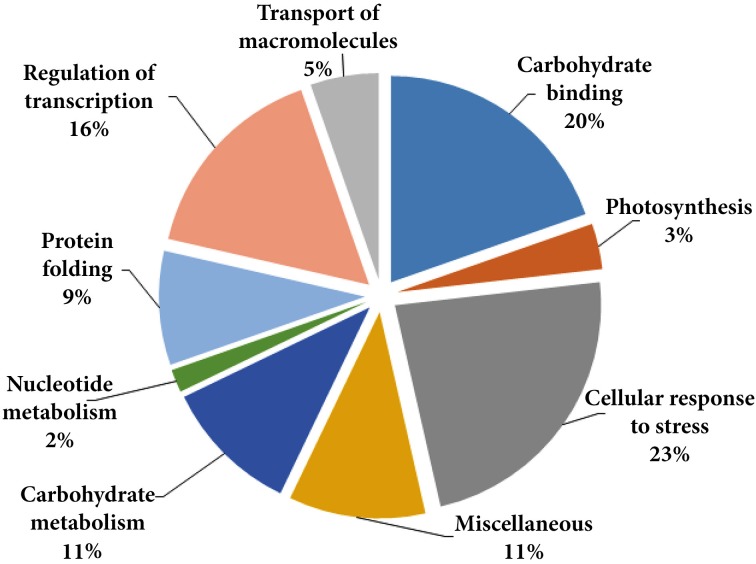
**Classification of the identified proteins, the pie chart shows the distribution of the low-temperature conditioning responsive proteins into their biological process in percentage according to Gene Ontology (http://www.geneontology.org/)**.

Considering the number of proteins identified and according to Gene Ontology these were grouped into nine different categories according to the biological processes in which they are involved (Table 1, Figure 2): cellular response to stress, carbohydrate binding, regulation of transcription, transport of macromolecules, protein folding, photosynthesis, carbohydrate metabolism, nucleotide metabolism, and miscellaneous. Many of the low-temperature conditioning-responsive proteins were isoforms with a change in p*I* and/or molecular weight strongly suggesting that the low-temperature conditioning-induced posttranslational modification and translation from alternatively spliced mRNAs of the candidate proteins, potentially including isoforms of multigenic families of proteins.

### Cellular response to stress related-proteins

Thirteen of the differentially accumulated protein spots were classified as related to cellular response to stress (Table 1). Spot 6, which decreased under low-temperature conditioning, was identified as the macrophage migration inhibitory factor (MIF) homolog isoform X3. In animal tissues MIF has been assigned different functions including cytokine activity, hormonal peptide, growth stimulant and extracellular oxidoreductase showing phenylpyruvate tautomerase, and dopachrome tautomerase activities (Kudrin and Ray, 2008). Whereas in plant tissues, MIF has been associated with defense mechanisms (Reumann et al., 2007) and as cytokine. Cytokines are secreted by stem cells and regulate plant immunity through cell-cell communication and reprogramming of the expression of immunity related genes (Luo, 2012). Cytokines not only enhance the innate immunity but also regulate division and differentiation of shoot apical meristem cells (Lee et al., 2011). However, the functional role of MIF could be more complex. Previous studies have indicated that MIF binds other proteins as Jab1 (Jun-activation-domain binding Protein) and CSN (COP9 signalosome), which are important in signaling pathways, as well as positive regulators of the cell cycle and protein degradation (Kleemann et al., 2000 and Bech-Otschir et al., 2002). The low accumulation of this protein could be related with the lower cell number and higher cell size in garlic bulbs from plants obtained from “seed” cloves stored 30 days at 4°C (Rahim and Fordham, 1988) as well as the lower height observed for our group when plants were conditioned at 5°C (Dufoo-Hurtado et al., 2013; Guevara-Figueroa et al., 2015).

Spots 17 and 18, with similar mass and p*I*, were identified as cystathionine β-synthase domain-containing protein (CBS); which showed opposite accumulation patterns that could indicate the same protein with some posttranslational modifications. Kushwaha et al. (2009) identified 34 CBS domain-containing proteins (CDCPs) in *Arabidopsis* and 59 in *Oryza* indicating that the CBS domain coexists with other functional domains. In addition, it was reported that CBS are differentially accumulated under cold stress conditions. Yoo et al. (2011) showed in *Arabidopsis thaliana* that proteins consisting of a single CBS domain pair stabilize cellular redox homeostasis and modulate plant development via regulation of ferredoxin-thioredoxin system (FTS). These CDCPs activate the thioredoxin in the FTS in chloroplast or in NADP-thioredoxin system in the mitochondria and thereby controls H_2_O_2_ levels. These authors showed that mutants with *35S:CBSX1* gene overexpressed had defective lignin deposition indicating that these CBS proteins affect the plant growth. Taking into account these observations, the over accumulation of one CBS protein in 5°C sprouts could indicate alterations in lignin deposition which will modify the plant growth that was reported by our group (Dufoo-Hurtado et al., 2013; Guevara-Figueroa et al., 2015).

Plant glutathione S-transferases (GSTs) have also long been associated with responses to biotic and abiotic stress, plant development and metabolism. Six spots (spots 21–26) that were down-accumulated in garlic sprouts in response to low-temperature conditioning were identified as GSTs. GST super-family is categorized into evolutionarily distinct classes. One of them is dehydroascorbate reductase (DHAR) class (spots 21–24), which is responsible for regenerating the ascorbic acid from an oxidized state maintaining the cellular ascorbic redox state allowing a greater tolerance to reactive oxygen species and better cell performance. The down-accumulation of this DHAR in samples conditioned at 5°C suggests an early oxidative stress, which could modify the plant development on the field. Chen and Gallie (2006) showed that suppression of DHAR expression in tobacco plants induced lower plant height and number of leaves, reduction of photosynthetic function, and premature leaf aging in comparison with control plants. The low leaves number and low width leaves as well as the lower height and weight of garlic plants from bulbs conditioned at 5°C observed for our group (Dufoo-Hurtado et al., 2013; Guevara-Figueroa et al., 2015) could be partially explained by the lower accumulation of these proteins. GST proteins also are involved in the synthesis of sulfur-containing secondary metabolites such as volatiles and glucosinolates, as well as in the conjugation, transport and storage of reactive oxylipins, phenolics, and flavonoids (Dixon and Edwards, 2010). The largest proportion of GSTs proteins in RT samples may also indicate more thiosulfinates synthesis (garlic aroma compounds) compared to samples from conditioning at 5°C. On the other hand, GST proteins also are required as cytoplasmic carrier proteins binding to flavonoids in order to deliver them into vacuoles and maintain the flavonoid pool under stress conditions (Kitamura, 2006). GSTs proteins in the samples from low-temperature conditioning could be involved in an active transport of anthocyanin and phenolic compounds into vacuoles, which increased in garlic bulbs obtained from cloves conditioned at low-temperature (Dufoo-Hurtado et al., 2013).

Increased accumulation of S-adenosylmethionine synthase (SAMS; spots 38, 45, and 61) in garlic sprouts under cold conditioning was observed. SAMS catalyzes the production of S-adenosyl-L-methionine (SAM) from L-methionine and ATP. SAM is a precursor of ethylene and polyamines whose levels significantly rise upon cold (Kosová et al., 2011) in addition, SAM is also a methyl donor potentially regulating recovery-related DNA/protein methylation and derivatives of the phenylpropanoid pathway (Cui et al., 2005; Chen et al., 2013). Enhanced accumulation of SAMS has been reported in response to cold stress (Cui et al., 2005; Amme et al., 2006; Yan et al., 2006; Chen et al., 2013). The enhanced accumulation of SAMS protein induced by the cold conditioning could be linked to an enhanced expression of the phenylpropanoid and flavonoid pathways genes in garlic tissues, which has also been previously reported by our group (Dufoo-Hurtado et al., 2013).

Spot 53 was identified as probable protein Pop3 showing an increased accumulation in response to low-temperature conditioning. Pop3 proteins represent a new class of proteins involved in the plant response to abiotic stress. They are hydrophilic like late embryogenesis abundant (LEA)-type proteins, and exhibit the oligomeric structure of heat shock proteins (HSPs; Wang et al., 2002). It has been shown that cold stress induced the accumulation of Pop3 (Renaut et al., 2005).

### Carbohydrate-binding proteins

Lectins are carbohydrate-binding proteins that specifically recognize and reversibly bind to specific free sugars or glycans present on glycoproteins and glycolipids without altering the structure of the carbohydrate and mediate a variety of biological processes (Lannoo and Van Damme, 2010). It has also been described that garlic mannose-specific lectins are the second most abundant proteins present in garlic cloves (Rabinkov et al., 1995). Ten spots (spots 3–5, 7–11, 51, and 54) were identified as garlic lectins, only one spot (spot 9) increased under cold conditioning, whereas the other nine spots decreased significantly. These results may indicate that cold conditioning of “seed” cloves could alter the metabolism of glycoproteins and glycolipids and their lectins. On the other hand, spot 9 could be associated with a lectin stress as indicated by Lannoo and Van Damme (2010) and Komath et al. (2006). In the meanwhile, since a number of lectins have been isolated from storage tissues in plants, it has been speculated that lectins might serve as plant storage proteins being important sources of nitrogen. These proteins could be degraded during the development process or bound to some non-polar molecules such as growth factors promoting the regulation of cell division (Komath et al., 2006). Therefore, a lower accumulation of these proteins during cold conditioning of “seed” garlic cloves at 5°C could be related with the less height, less number and width of leaves reported by Dufoo-Hurtado et al. (2013) and Guevara-Figueroa et al. (2015).

### Proteins related to regulation of transcription

Nine of the differentially accumulated protein spots were classified as related to regulation of transcription (Table 1). Among them, only one spot (spot 39) decreased under cold conditioning and was identified as plasminogen activator inhibitor 1 RNA-binding protein-like protein (PAI1). On the contrary, eight spots were significantly increased in response to cold conditioning in garlic sprouts. Five spots (spots 13–16, and 55) were identified as glycine-rich RNA-binding proteins (GR-RBPs). One spot (spot 33) was identified as serine/arginine-rich splicing factor (SR) involved in splicing of pre-mRNA and stress responses (Reddy and Shad Ali, 2011). Spot 36 was identified as heterogeneous nuclear ribonucleoprotein (hnRNP) that could be involved in mRNA transport (Kawamura et al., 2002). Spot 59 was identified as polyadenylate-binding protein (PAPB), which plays critical roles in eukaryotic translation initiation and mRNA stabilization/degradation (Deo et al., 1999). These proteins (GR-RBPs, SR, hnRNP, and PAPB) are characterized by the presence of RNA-recognition motifs (RRMs) and are described as RNA-binding proteins (RBPs). Nowadays there is evidence that suggests that RNA-binding proteins are involved in a series of abiotic stresses responses, including cold adaptation (Kim et al., 2007). The RNA-binding activity of those proteins has been biochemically demonstrated, suggesting that they might be involved in the regulation of pre-mRNA alternative splicing, mRNA export, mRNA translation, mRNA decay, long term storage of “masked messages,” and perhaps even mRNA localization. These functions surely facilitate a more rapid and effective response to a severe biotic and abiotic stress (Mangeon et al., 2010; Ciuzan et al., 2015). It is important to note that the changes in these regulatory proteins are expressed in meristematic tissue of “seed” cloves during the cold conditioning and those changes will have a significant effect during plant development.

### Proteins related to transport of macromolecules

Nascent polypeptide-associated complex subunit (NAC, spot 27) was significantly accumulated after cold conditioning. NAC proteins bind to newly synthesized polypeptide chains from ribosome to protect them from proteolysis and to facilitate their folding. Through interaction with signal recognition particles, NAC also participates in the transport to endoplasmic reticulum of newly synthetized proteins, but there also exist evidence that this protein shifts from the soluble to the insoluble aggregated protein fraction during cell aging (Park et al., 2012). This protein acts as a modulator of protein synthesis and it is a key regulator of protein homeostasis (proteostasis) during the response to abiotic stress (Kirstein-Miles et al., 2013; Kogan and Gvozdev, 2014). On the other hand, it has also been reported that silencing of the NAC gene induced tolerance to freezing and drought stress exposures in wheat (Kang et al., 2013), which seems to be different in garlic sprouts.

Spot 34 decreased in abundance in cold conditioning samples and it was identified as annexin D2-like (ANX2). Annexins are soluble proteins and are defined as multifunctional Ca^2+^ and lipid-binding proteins. Plant annexins are expressed throughout the life cycle and have been detected in all organs. It is estimated that annexins can comprise 0.1% of plant cell protein. Plant annexin expression and localization appear linked to growth and development (Laohavisit and Davies, 2011). It has been reported that cold stress causes increased accumulation of annexin in poplar leaves (Renaut et al., 2006) but in this study the accumulation of annexin protein was lower in cold conditioning samples than RT samples, which could indicate other functions in these tissues. Mortimer et al. (2008) indicated that they could be central regulators or effectors of plant growth and stress signaling. Potentially, they could relocate to membranes as response to reactive oxygen species and cytosolic free calcium to cope with adverse conditions such as cold. Furthermore, annexins not only participate in the regulation of membrane organization and vesicle trafficking, they also seem to be involved in Ca^2+^-regulated exocytosis or some endocytic events. Lannoo and Van Damme (2010) reported that annexins are involved in RNA-binding, sensing or transducing Ca^2+^ signals or in the regulation of [Ca^2+^]_cyt_ during signaling process.

Transport of molecules with high molecular weight between the cytoplasm and the nucleoplasm requires an active mechanism facilitated by soluble nuclear transport factors or NTFs (Zhou et al., 2013). Spot 54 identified as NTF2 significantly decreased in abundance after cold conditioning. NTF2 is one of several essential components of Ran cycle involved in nuclear trafficking. Its main role is to transport RanGDP from cytoplasm to nucleus and replenish the nuclear pools of RanGTP (Bian et al., 2011). It has been observed that overexpression of NTF2 inhibits nuclear import of transcription factors, some of which are involved in stress-dependent gene induction (Zhao et al., 2006; Bian et al., 2011; Binder and Parniske, 2013).

### Proteins related to protein folding

The risk of protein misfolding increases at low temperatures, resulting in non-functional proteins. For that reason, a higher accumulation of proteins with chaperone functions has been reported under low temperature conditions (Renaut et al., 2004, 2005; Cui et al., 2005; Yan et al., 2006; Rinalducci et al., 2011; Chen et al., 2013). Five spots of proteins differentially accumulated were identified as proteins with chaperone functions. Three spots significantly increased in response to cold conditioning; spot 41 was identified as chaperonin CPN60-2, spot 43 was identified as heat shock 70 kDa protein (HSP70) and the spot 62 was identified as peptidyl-prolyl cis-trans isomerase FKBP12 (PPIase). According to Mühlhaus et al. (2011) the presence of HSPs in protein aggregates facilitates the access of ATP-dependent chaperones ClpB and Hsp70/Hsp40, which catalyze the refolding of the complex, denatured proteins back to its functional state. However, the increased accumulation of chaperones may play a pivotal role in preventing aggregation and in facilitating the refolding of denatured proteins under normal and low-temperature conditions. In contrast, two spots decreased under cold conditioning were identified as a chaperone (spot 19) and 18.1 kDa class I heat shock protein (HSP18.1; spot 20) but the specific role of these proteins in folding or refolding proteins is not known.

### Photosynthesis-related proteins

In garlic sprouts, we found two spots identified as small subunits of chloroplast ribulose-1,5-bisphosphate carboxylase/oxygenase (RuBisCO SSU; spots 12 and 62) that showed an interestingly contrasting accumulation. Low temperature affects significantly photosynthesis, but plants may adjust photosynthesis via gene regulation to adapt to cold environment and some proteins involved in Calvin cycle and electron transport. It has been shown that RuBisCO SSUs are important for catalysis by enhancing the catalytic rate and specificity through inducing conformational changes in RuBisCO large subunits (Spreitzer, 2003). Yoom et al. (2001) found that the relative SSU gene expression differed significantly between plants grown at different temperatures showing that some genes were over expressed in cold conditions (5 and 10°C) and others increased at high temperature. In leaves of *Thellungiella* exposure to cold conditions (24 h at 4°C) increased the accumulation of two SSU proteins, while other SSU decreased, whereas the RuBisCO large subunit decreased (Gao et al., 2009). These data suggested that garlic plants might modify their photosynthetic efficiency and could explain the slower growth rate of the plants observed at the end of the crop cycle. Although other explanation could be, as in the case of the photosystem II that was being photo-inhibited because the photorespiration pathway (a protective mechanism of photosynthetic systems in C_3_ plants) could be enhanced by low temperature, which also would reduce the CO_2_ fixation (Zhang et al., 2013) causing lower plant growth.

### Carbohydrate metabolism-related proteins

Different studies have indicated changes in abundance of enzymes involved in carbohydrate metabolism under low temperature conditions. In general, up-regulation of catabolic pathways and down-regulation of anabolic pathways has been observed under cold stress (Kosová et al., 2011). Accumulation of tricarboxylic acid (TCA) cycle enzymes could also suggest an efficient recycling of amino acids as energy source and their subsequent recruitment as substrates in other cellular pathways under low temperature exposure (Rinalducci et al., 2011). Our data showed significant increased accumulation of carbohydrate metabolism-related proteins in six protein spots from garlic sprouts under cold conditioning (Figure 1, Table 1). Fructose-bisphosphate aldolase (FBPA; spot 37) and 2,3-bisphosphoglycerate-independent phosphoglycerate mutase (iPGAM; spots 42 and 46). FBPA catalyzes the conversion of D-fructose 1,6-bisphosphate into dihydroxyacetone phosphate and D-glyceraldehyde 3-phosphate. This enzyme plays an important role in carbohydrate metabolism and in the production of triose phosphate derivatives, which are important in signal transduction (Schaeffer et al., 1997). iPGAM besides to its role in glycolysis, also plays an important role in the biosynthesis of aromatic amino acids and aromatic compounds (Zhao and Assmann, 2011). Whereas malate dehydrogenase (MDH), detected in spots 35, 37, and 44, is involved in the TCA cycle as well as in glyoxylate bypass, amino acid synthesis, gluconeogenesis and as a shuttle between cytoplasm and subcellular organelles (Musrati et al., 1998). The up-regulation of carbohydrate metabolism might help in more energy production or intermediate compounds needed in cell adaptation to the stress condition.

### Nucleotide metabolism-related proteins

Nucleotide metabolism was altered under cold conditioning as revealed by the increased accumulation of adenosine kinase 2 (ADK2; spot 33). ADK is a purine kinase that transfer γ-phosphate from ATP to adenosine to generate 5′-AMP (Mohannath et al., 2014). This enzyme plays an important role in the adenine salvage pathway and thereby contributes to the maintenance of cellular energy and in the synthesis of different biomolecules including nucleotide cofactors and nucleic acids. ADK has also other roles in plant metabolism; it is associated to maintain methyl transferase activities by sustaining the methyl cycle that generates SAM. Second, is involved in the mechanism for regulating the level of active cytokinin in plants (Moffatt et al., 2002). It has been established that adenosine kinase forms a complex with SNF1-related kinase (SnRK1) in plants and that these complexes may play important roles in energy homeostasis and cellular responses to biotic and abiotic stress (Mohannath et al., 2014).

### Miscellaneous proteins

Six different spots (Table 1) were identified as the coat protein (CP) of *garlic common latent virus* (GarCLV). Five proteins were decreased in abundance under cold conditioning (spots 31, 32, 47, 49 and 57) and another was increased in abundance (spot 33). GarCLV has been reported in different countries around the world and it is always affecting garlic crop. However, this virus is symptomless on garlic but can induce severe yellowing and mosaic when it occurs in mixture with other viruses (Pramesh and Baranwal, 2013). The decrease in the accumulation of virus coat proteins may be a response of the virus related to environmental conditions (Parrano et al., 2012).

## Concluding remarks

**Figure 3:**
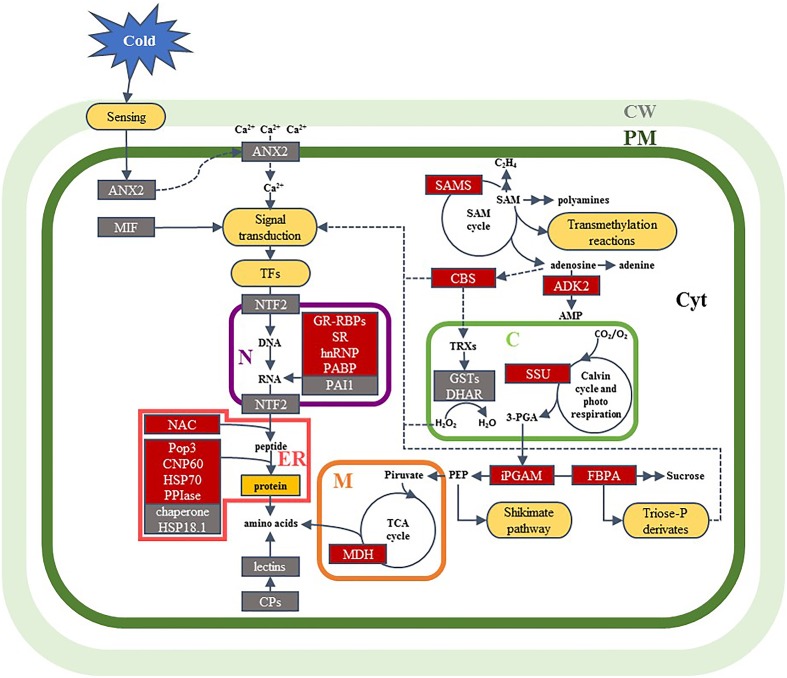
**A model summarizing of the effects of cold conditioning into a garlic sprout cell derived from the changes observed at proteome level**. Identified proteins are shown in boxes; red boxes, proteins that increased in abundance in response to the low-temperature conditioning; gray boxes, proteins that decreased in abundance in response to cold conditioning. Abbreviations for cell components: C, chloroplast; CW, cell wall; Cyt, cytoplasm; ER, endoplasmic reticulum; M, mitochondria; N, nucleus; PM, plasma membrane. Abbreviations for metabolites, enzymes and other proteins: 3-PGA, 3-phosphoglyceric acid; ADK2, adenosine kinase 2-like; AMP, adenosine monophosphate; ANX2, annexin D2-like; CBS, cystathionine β-synthase domain protein; CPN60, chaperonin CPN60-2; CP, coat protein; DHAR, dehydroascorbate reductase; FBPA, fructose-bisphosphate aldolase; GR-RBP, glycine-rich RNA-binding protein; GST, glutathione S-transferase; hnRNP, heterogeneous nuclear ribonucleoprotein 1-like; HSP18.1, 18.1 kDa class I heat shock protein-like; HSP70, heat shock 70 kDa protein; iPGAM, 2,3-bisphosphoglycerate-independent phosphoglycerate mutase; MDH, malate dehydrogenase; MIF, macrophage migration inhibitory factor homolog; NAC, nascent polypeptide-associated complex subunit; NTF2, nuclear transport factor 2-like; PABP, polyadenylate-binding protein RBP45-like; PAI1, plasminogen activator inhibitor 1 RNA-binding protein-like; PEP, phosphoenol pyruvate; Pop3, probable protein Pop3; PPIase, peptidyl-prolyl cis-trans isomerase FKBP12; SAM, S-adenosyl-L-methionine; SAMS, S-adenosyl-L-methionine synthase; SR, serine/arginine-rich splicing factor SR34A-like; SSU, RuBisCO small subunit; TCA, tricarboxylic acid; TF, transcription factor; TRX, thioredoxin.

Based on the previous discussion, a hypothetical model is proposed to illustrate cellular events potentially associated with the effects of low-temperature conditioning (Figure 3). We have shown that cold conditioning induces down-accumulation of ANX2, due to a temporary relocation to membranes in response to cold. This situation could change the [Ca^2+^]_*cyt*_ inducing a different signaling process. The signal is transmitted to the cellular machinery by signal transduction and transcription factors that regulate the gene expression and protein accumulation to establish a new cellular homeostasis. In this sense, the decrease of NTF2 could lead to an increase in the nuclear import of transcription factors, some of which are involved in stress-dependent gene induction. These responses could explain the accumulation of proteins related with the regulation of transcription, RNA processing, translation, and protein processing, such as PAI1, GR-RBPs, SR, hnRNP, PAPB, and NAC. In addition, protein-folding proteins increased in garlic sprouts in response to the cold conditioning, such as CPN60-2, HSP70, PPIase and Pop3 that stabilize proteins and help to restore the cellular proteostasis. Several key enzymes regulating carbohydrate metabolism (and ATP production) accumulation in cold conditioned samples (iPGAM, FBPA, MDH) might help in energy production or intermediate compounds needed in cell adaptation to the low temperature environment. We have found the accumulation of ADK2 in cold conditioned samples, which contributes to energy homeostasis and in the synthesis of nucleotide cofactors and nucleic acids. ADK2 is associated with the methyl cycle that generates SAM, a methyl donor involved in secondary metabolism; in addition, increased accumulation of SAMS in cold conditioned samples was observed. In another hand, the decrease in the abundance of proteins such as MIF, CBS, DHAR and lectins, which have key roles in the redox homeostasis, lignin deposition, division and differentiation of cells, could explain the differences in plant growth. Our results also showed differences in the abundance of RuBisCO SSU related with photorespiration that reflects the importance of this pathway as a protective mechanism of photosynthetic systems in C_3_ plants, which also reduces the CO_2_ fixation causing lower plant growth. Reduced accumulation of GST proteins in 5°C samples could indicate an increased accumulation of H_2_O_2_. Redox homeostasis and glycolysis metabolites can also act as signals and promote the regulation of gene expression. Conceivably, further studies will provide additional information for this model.

The data presented in this research indicate that the low-temperature conditioning of “seed” garlic cloves during 5 weeks at 5 °C affected different metabolic pathways and physiological processes. The physiological processes affected include cellular growth, antioxidative/oxidative state, macromolecules transport, protein folding, and transcription regulation process. The metabolic pathways affected including protein biosynthesis and quality control system, photosynthesis, photorespiration, energy production, and carbohydrate and nucleotide metabolism. These processes can work cooperatively to establish a new cellular homeostasis that might be related with the physiological and biochemical changes observed in previous studies (Dufoo-Hurtado et al., 2013; Guevara-Figueroa et al., 2015). This is the first work that reports the changes in the protein profiles of garlic “seed” cloves to low-temperature conditioning. The identification of cold-responsive proteins in garlic provides not only new insights into cold conditioning responses but also a good starting point for further dissection of their functions during the development of the crop using genetic and other approaches.

## Author contributions

MD, EM, JH, and AB, conceived the study and designed the 2-DE experiments. MD and JH performed the experiments. AB performed the LC-MS/MS analysis. MD and JH carried out the analysis of the data, made the identification of the proteins and drafted the manuscript. EM and AB contributed in the preparation of the final draft of the manuscript. AB provided reagents, materials and analysis tools. All authors (MD, JH, AB, EM, and AB) read and approved the final manuscript.

### Conflict of interest statement

The authors declare that the research was conducted in the absence of any commercial or financial relationships that could be construed as a potential conflict of interest.

## Abbreviations

ADK2: Adenosine kinase 2
ANX2: Annexin D2-like
CBS: Cystathionine β-synthase
CP: Coat protein
DHAR: Dehydroascorbate reductase
FBPA: Fructose-bisphosphate aldolase
FTS: Ferredoxin-thioredoxins system
GarCLV: *Garlic common latent virus*
GR-RBP: Glycine-rich RNA-binding protein
GST: Glutathione S-transferase
hnRNP: Heterogeneous nuclear ribonucleoprotein
HSP: Heat shock protein
iPGAM: 2,3-bisphosphoglycerate-independent phosphoglycerate mutase
LEA: Late embryogenesis abundant
MDH: Malate dehydrogenase
MIF: Macrophage migration inhibitory factor
NAC: Nascent polypeptide-associated complex
NTF: Nuclear transport factors
PAI1: Plasminogen activator inhibitor 1 RNA-binding protein-like protein
PAPB: Polyadenylate-binding protein
PPIase: Peptidyl-prolyl cis-trans isomerase
RRM: RNA-recognition motif
RT: Conditioning at room temperature
RuBisCO: Ribulose-1,5-bisphosphate carboxylase/oxygenase
SAM: S-adenosyl-L-methionine
SAMS: S-adenosylmethionine synthase
SnRK1: SNF1-related kinase
SR: Serine/arginine-rich splicing factor
SSU: RuBisCO small subunit
TCA: Tricarboxylic acid cycle.
