# Supplementary material for: Low temperature conditioning of garlic (Allium sativum L.) “seed” cloves induces alterations in sprouts proteome
Source: Front Plant Sci. 2015 May 13;6:332. doi: 10.3389/fpls.2015.00332 (PMC4429546; doi:10.3389/fpls.2015.00332)
Supplement: Supplementary file 1 [file DataSheet1.DOCX]

**Low temperature conditioning of garlic (*Allium sativum L.*) “seed” cloves induces alterations in sprouts proteome**

Miguel David Dufoo-Hurtado^1^, José Ángel Huerta-Ocampo^2,+^, Alberto Barrera-Pacheco^2^, Ana Paulina Barba de la Rosa^2^, Edmundo Mercado-Silva^1^,

^1^ Laboratorio de Fisiología y Bioquímica Poscosecha de Frutas y Hortalizas, Departamento de Investigación y Posgrado, Facultad de Química, Universidad Autónoma de Querétaro, Querétaro, Querétaro, México.

^2^ Laboratorio de Proteómica y Biomedicina Molecular, División de Biología Molecular, Instituto Potosino de Investigación Científica y Tecnológica A.C., San Luis Potosí, San Luis Potosí, México.

^+^ Actual address: Laboratorio de Bioquímica de Proteínas y Glicanos, Coordinación de Ciencia de los Alimentos, Centro de Investigación en Alimentación y Desarrollo A.C., Hermosillo, Sonora, México.

***Correspondence:**

Edmundo Mercado Silva

Universidad Autónoma de Querétaro

Facultad de Química

Departamento de Investigación y Posgrado

Laboratorio de Fisiología y Bioquímica Poscosecha de Frutas y Hortalizas

Cerro de las Campanas s/n. Col. Las Campanas,

Querétaro, Querétaro, 76010, México

mercado501120@gmail.com; mercasilva20@yahoo.com.mx

**Supplementary Figures**


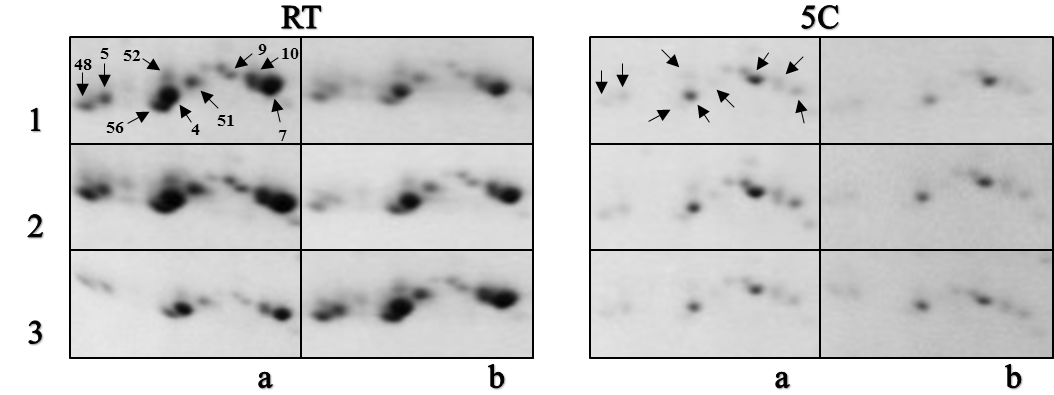


**Figure S1**. Zoomed images showing the region of protein spots 4, 5, 7, 9, 10, 48, 51, 52, and 56, between samples at room temperature (RT) or low-temperature conditioning (5C), as an example of the reproducibility of the gels. Two technical replicates (columns a, and b) per each of the three biological replicates (rows 1, 2, and 3) were run and analyzed by Melanie v 7.0 software.


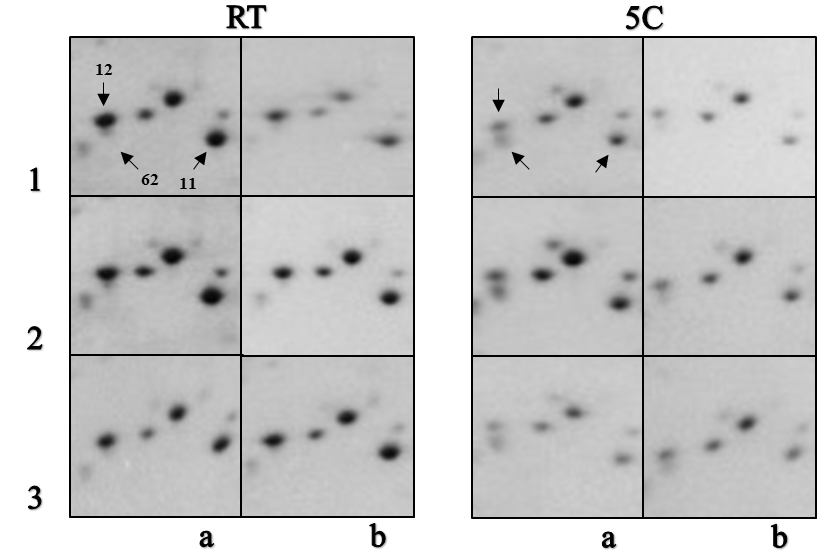


**Figure S2**. Zoomed images showing the region of protein spots 11, 12, and 62, between samples at room temperature (RT) or low-temperature conditioning (5C), as an example of the reproducibility of the gels. Two technical replicates (columns a, and b) per each of the three biological replicates (rows 1, 2, and 3) were run and analyzed by Melanie v 7 software.


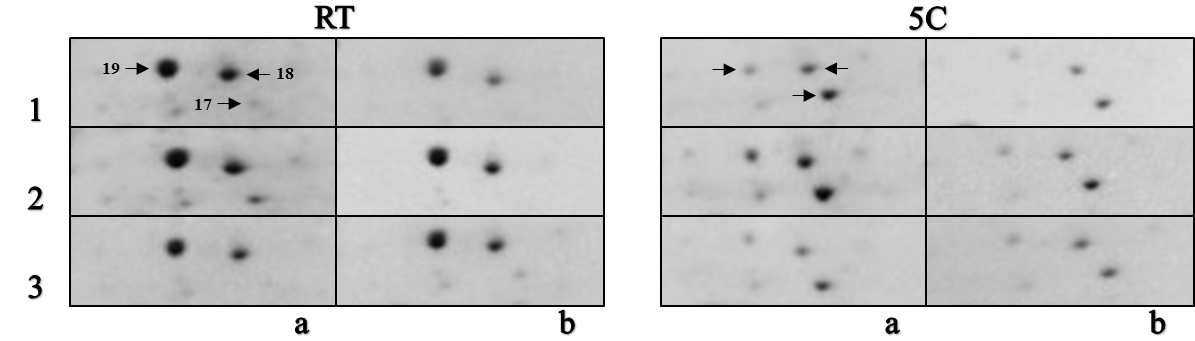


**Figure S3**. Zoomed images showing the region of protein spots 17, 18, and 19, between samples at room temperature (RT) or low-temperature conditioning (5C), as an example of the reproducibility of the gels. Two technical replicates (columns a, and b) per each of the three biological replicates (rows 1, 2, and 3) were run and analyzed by Melanie v 7.0 software.
