# Supplementary material for: Low temperature conditioning of garlic (Allium sativum L.) “seed” cloves induces alterations in sprouts proteome
Source: Front Plant Sci. 2015 May 13;6:332. doi: 10.3389/fpls.2015.00332 (PMC4429546; doi:10.3389/fpls.2015.00332)
Supplement: Supplementary file 2 [file DataSheet2.DOCX]

**Low temperature conditioning of garlic (*Allium sativum L.*) “seed” cloves induces alterations in sprouts proteome**

Miguel David Dufoo-Hurtado^1^, José Ángel Huerta-Ocampo^2,+^, Alberto Barrera-Pacheco^2^, Ana Paulina Barba de la Rosa^2^, Edmundo Mercado-Silva^1*^

^1^ Laboratorio de Fisiología y Bioquímica Poscosecha de Frutas y Hortalizas, Departamento de Investigación y Posgrado, Facultad de Química, Universidad Autónoma de Querétaro, Querétaro, Querétaro, México.

^2^ Laboratorio de Proteómica y Biomedicina Molecular, División de Biología Molecular, Instituto Potosino de Investigación Científica y Tecnológica A.C., San Luis Potosí, San Luis Potosí, México.

^+^ Actual address: Laboratorio de Bioquímica de Proteínas y Glicanos, Coordinación de Ciencia de los Alimentos, Centro de Investigación en Alimentación y Desarrollo A.C., Hermosillo, Sonora, México.

***Correspondence:**

Edmundo Mercado Silva

Universidad Autónoma de Querétaro

Facultad de Química

Departamento de Investigación y Posgrado

Laboratorio de Fisiología y Bioquímica Poscosecha de Frutas y Hortalizas

Cerro de las Campanas s/n. Col. Las Campanas,

Querétaro, Querétaro, 76010, México

mercado501120@gmail.com; mercasilva20@yahoo.com.mx

**Supplementary Table**

**Table S1**. Identification of differentially accumulated proteins in *Allium sativum* L. sprouts subjected to low-temperature conditioning by LC-ESI-MS/MS analysis.

| **Spot number ^a)^** | **Protein name** | **Plant species ^(b)^** | **NCBInr accession number ^(c)^** | **Exper. kDa/p*I* ^(d)^** | **Theor. kDa/p*I* ^(e)^** | **Mascot score ^(f)^** | **PM ^(g)^** | **SC ^(h)^** | **Peptide sequence** | **Observed mass (m/z)** | **Charge** | **Matched mass (calculated)** | **Delta mass (ppm)** | **DB ^(i)^** | **As trans accession number ^(j)^** | **Fr ^(k)^** | **E-value ^(l)^** | **BP ^(m)^** | **Fold change ^(n)^** | **V%+SE ^(o)^** | |
| --- | --- | --- | --- | --- | --- | --- | --- | --- | --- | --- | --- | --- | --- | --- | --- | --- | --- | --- | --- | --- | --- |
|  |  |  |  |  |  |  |  |  |  |  |  |  |  |  |  |  |  |  |  | **RT** | **5C** |
| 1 | Not identified | *---* | --- | 9.0/5.36 | --- | --- | --- | --- | --- | --- | --- | --- | --- | --- | --- | --- | --- | --- | 2.81 |  | |
| 2 | Not identified | *---* | --- | 11.0/5.66 | --- | --- | --- | --- | --- | --- | --- | --- | --- | --- | --- | --- | --- | --- | 0.54 |  | |
| 3 | Mannose-specific lectin | *Allium sativum* | gi\|1840047 | 11.0/5.05 | 19.5/8.84 | 181 | 2 | 18% | R.DGNFVVYDVNGRPVWASNSVR.G | 784.3858 | 3+ | 2350.1455 | -4.2 | NCBI | --- | -- | --- | 2 | 0.48 |  | |
|  |  |  |  |  |  |  |  |  | R.GNGNYILLLQEDR.N | 752.8936 | 2+ | 1503.7681 | 3.0 |  |  |  |  |  |  |  |  |
| 4 | Chain A, Mannose-Specific Agglutinin (Lectin) | *Allium sativum* | gi\|4389040 | 10.0/4.70 | 11.85/5.12 | 184 | 2 | 24% | K.AVLQSDGNFVVYDAEGR.S | 920.4509 | 2+ | 1838.8799 | 4.0 | NCBI | --- | -- | --- | 2 | 0.30 |  | |
|  |  |  |  |  |  |  |  |  | R.SLWASHSVR.G | 521.7739 | 2+ | 1041.5356 | -2.2 |  |  |  |  |  |  |  |  |
| 5 | Chain A, Mannose-Specific Agglutinin (Lectin) | *Allium sativum* | gi\|4389040 | 10.0/4.55 | 11.85/5.12 | 42 | 2 | 24% | K.AVLQSDGNFVVYDAEGR.S | 920.4471 | 2+ | 1838.8799 | -0.1 | NCBI | --- | -- | --- | 2 | 0.28 |  | |
|  |  |  |  |  |  |  |  |  | R.SLWASHSVR.G | 521.7769 | 2+ | 1041.5356 | 3.5 |  |  |  |  |  |  |  |  |
| 6 | Macrophage migration inhibitory factor homolog isoform X3 | *Brassica rapa* | gi\|685279434 | 11.0/5.53 | 12.1/5.24 | 118 | 2 | -- | K.DLSAAVSSILESK.L | 660.3624 | 2+ | 1318.6980 | 9.0 | As | gi\|378366384 | 1 | 9E-54 | 1 | 0.54 |  | |
|  |  |  |  |  |  |  |  |  | K.FYGAEGSFMGWNGSTF.- + Oxidation (M9) | 887.3666 | 2+ | 1772.7141 | 3.0 |  |  |  |  |  |  |  |  |
| 7 | Chain A, Mannose-Specific Agglutinin (Lectin) | *Allium sativum* | gi\|4389040 | 11.0/4.94 | 11.85/5.12 | 58 | 2 | 24% | K.AVLQSDGNFVVYDAEGR.S | 920.4494 | 2+ | 1838.8799 | 2.4 | NCBI | --- | -- | --- | 2 | 0.16 |  | |
|  |  |  |  |  |  |  |  |  | R.SLWASHSVR.G | 521.7772 | 2+ | 1041.5356 | 4.1 |  |  |  |  |  |  |  |  |
| 8 | Chain A, Mannose-Specific Agglutinin (Lectin) | *Allium sativum* | gi\|4389040 | 11.0/5.20 | 11.85/5.12 | 53 | 2 | 24% | K.AVLQSDGNFVVYDAEGR.S | 920.4531 | 2+ | 1838.8799 | 6.4 | NCBI | --- | -- | --- | 2 | 0.51 |  | |
|  |  |  |  |  |  |  |  |  | R.SLWASHSVR.G | 521.7714 | 2+ | 1041.5356 | -7.0 |  |  |  |  |  |  |  |  |
| 9 | Chain A, Mannose-Specific Agglutinin (Lectin) | *Allium sativum* | gi\|4389040 | 11.0/4.85 | 11.85/5.12 | 72 | 2 | 24% | K.AVLQSDGNFVVYDAEGR.S | 920.4485 | 2+ | 1838.8799 | 1.4 | NCBI | --- | -- | --- | 2 | 2.34 |  | |
|  |  |  |  |  |  |  |  |  | R.SLWASHSVR.G | 521.7774 | 2+ | 1041.5356 | 4.5 |  |  |  |  |  |  |  |  |
| 10 | Chain A, Mannose-Specific Agglutinin (Lectin) | *Allium sativum* | gi\|4389040 | 11.0/4.90 | 11.85/5.12 | 100 | 2 | 24% | R.AVLQSDGNFVVYDAEGR.S | 920.4477 | 2+ | 1838.8799 | 0.5 | NCBI | --- | -- | --- | 2 | 0.42 |  | |
|  |  |  |  |  |  |  |  |  | R.SLWASHSVR.G | 521.7742 | 2+ | 1041.5355 | -1.7 |  |  |  |  |  |  |  |  |
| 11 | Lectin | *Allium sativum* | gi\|102230895 | 13.0/6.49 | 19.4/9.05 | 232 | 3 | 26% | R.DGNFVVYDVNGRPVWASNSVR.G | 784.3892 | 3+ | 2350.1455 | 0.1 | NCBI | --- | -- | --- | 2 | 0.53 |  | |
|  |  |  |  |  |  |  |  |  | R.GNGNYILVLQK.D | 609.8475 | 2+ | 1217.6768 | 3.0 |  |  |  |  |  |  |  |  |
|  |  |  |  |  |  |  |  |  | R.NVVIYGSDIWSTGTYR.R | 915.9562 | 2+ | 1829.8948 | 1.6 |  |  |  |  |  |  |  |  |
| 12 | Chloroplast ribulose-1,5-bisphosphate carboxylase/oxygenase small subunit | *Musa acuminata* AAA Group | gi\|76574234 | 13.0/6.24 | 18.2/9.05 | 141 | 4 | -- | K.VWPIEGLK.K | 471.2748 | 2+ | 940.5382 | -3.0 | As | gi\|378345772 | 1 | 5E-46 | 6 | 0.58 |  | |
|  |  |  |  |  |  |  |  |  | K.VWPIEGLKK.F | 535.3259 | 2+ | 1068.6331 | 4.0 |  |  |  |  |  |  |  |  |
|  |  |  |  |  |  |  |  |  | K.KFETLSYLPPLTDEQLLK.Q | 712.3923 | 3+ | 2134.1561 | -1.0 |  |  |  |  |  |  |  |  |
|  |  |  |  |  |  |  |  |  | K.QIEYLIR.N | 467.7696 | 2+ | 933.5283 | -4.0 |  |  |  |  |  |  |  |  |
| 13 | RNA-binding glycine-rich protein-1b | *Nicotiana sylvestris* | gi\|469071 | 14.0/5.04 | 14.8/5.55 | 49 | 2 | 20% | R.DAIEGMNGQELDGR.S + Oxidation (M6) | 760.8373 | 2+ | 1519.6573 | 1.8 | NCBI | --- | -- | --- | 3 | 2.04 |  | |
|  |  |  |  |  |  |  |  |  | R.EGGGGGYGGGGYGGGGR.Y | 686.2889 | 2+ | 1370.5600 | 2.4 |  |  |  |  |  |  |  |  |
| 14 | Glycine-rich RNA-binding protein GRP1A-like | *Vitis vinifera* | gi\|359475330 | 14.0/5.22 | 16.3/6.32 | 388 | 6 | -- | K.VFSQFGEVVESK.I | 678.3457 | 2+ | 1354.6769 | 0.0 | As | gi\|378349200 | 6 | 2E-43 | 3 | 4.80 |  | |
|  |  |  |  |  |  |  |  |  | R.GFGFVTFNSEQSMR.E + Oxidation (M13) | 811.8674 | 2+ | 1621.7195 | 0.0 |  |  |  |  |  |  |  |  |
|  |  |  |  |  |  |  |  |  | R.EAIEGMNGQNLDGR.S + Oxidation (M6) | 760.3441 | 2+ | 1518.6732 | 0.0 |  |  |  |  |  |  |  |  |
|  |  |  |  |  |  |  |  |  | R.EGGGGGGYGQR.R | 497.7233 | 2+ | 993.4264 | 6.0 |  |  |  |  |  |  |  |  |
|  |  |  |  |  |  |  |  |  | R.EGGGGGYGGGGGGGGYNR.G | 743.3107 | 2+ | 1484.6029 | 3.0 |  |  |  |  |  |  |  |  |
|  |  |  |  |  |  |  |  |  | R.GGGGGYGGESGGSR.Y | 577.7470 | 2+ | 1153.4749 | 4.0 |  |  |  |  |  |  |  |  |
| 15 | Glycine-rich RNA-binding protein GRP1A-like | *Vitis vinifera* | gi\|359475330 | 15.0/5.46 | 16.3/6.32 | 405 | 7 | -- | R.CFVGGLAWATDDNALER.A | 947.9428 | 2+ | 1893.8680 | 2.0 | As | gi\|378347384 | 2 | 2E-44 | 3 | 3.03 |  | |
|  |  |  |  |  |  |  |  |  | R.AFSQFGEVVDSK.I | 657.3231 | 2+ | 1312.6299 | 1.0 |  |  |  |  |  |  |  |  |
|  |  |  |  |  |  |  |  |  | R.GFGFVTFSSEQSMR.D + Oxidation (M13) | 798.3639 | 2+ | 1594.7086 | 3.0 |  |  |  |  |  |  |  |  |
|  |  |  |  |  |  |  |  |  | R.DAIEGMNGQDLDGR.N + Oxidation (M6) | 753.8280 | 2+ | 1505.6416 | 0.0 |  |  |  |  |  |  |  |  |
|  |  |  |  |  |  |  |  |  | R.GGGGGGGGGFGGGQR.R | 567.7568 | 2+ | 1133.4963 | 2.0 |  |  |  |  |  |  |  |  |
|  |  |  |  |  |  |  |  |  | R.EGGGGGYGQR.R | 469.2108 | 2+ | 936.4050 | 2.0 |  |  |  |  |  |  |  |  |
|  |  |  |  |  |  |  |  |  | R.EGGGGGGYGGGGGYGR.S | 657.7797 | 2+ | 1313.5385 | 5.0 |  |  |  |  |  |  |  |  |
| 16 | Glycine-rich RNA-binding protein GRP1A-like | *Vitis vinifera* | gi\|359475330 | 16.0/4.90 | 16.3/6.32 | 112 | 4 | -- | R.AFSQFGEVVDSK.I | 657.3244 | 2+ | 1312.6299 | 3.0 | As | gi\|378347384 | 2 | 2E-44 | 3 | 1.84 |  | |
|  |  |  |  |  |  |  |  |  | R.GFGFVTFSSEQSMR.D + Oxidation (M13) | 798.3653 | 2+ | 1594.7086 | 5.0 |  |  |  |  |  |  |  |  |
|  |  |  |  |  |  |  |  |  | R.DAIEGMNGQDLDGR.N + Oxidation (M6) | 753.8301 | 2+ | 1505.6416 | 3.0 |  |  |  |  |  |  |  |  |
|  |  |  |  |  |  |  |  |  | R.GGGGGGGGGFGGGQR.R | 567.7561 | 2+ | 1133.4963 | 1.0 |  |  |  |  |  |  |  |  |
| 17 | CBS domain-containing protein | *Coffea canephora* | gi\|661892853 | 18.0/6.38 | 24.1/9.32 | 62 | 2 | -- | K.LITVNPETK.V | 507.7976 | 2+ | 1013.5757 | 4.9 | As | gi\|378356533 | 1 | 4E-117 | 1 | 6.06 |  | |
|  |  |  |  |  |  |  |  |  | R.HIPVIDNK.N | 468.2695 | 2+ | 934.5236 | 1.0 |  |  |  |  |  |  |  |  |
| 18 | CBS domain-containing protein | *Coffea canephora* | gi\|661892853 | 19.0/6.33 | 24.1/9.32 | 157 | 3 | -- | K.VLQAMELMTEK.R + Oxidation (M5) | 654.8272 | 2+ | 1307.6465 | -5.1 | As | gi\|378356533 | 1 | 4E-117 | 1 | 0.69 |  | |
|  |  |  |  |  |  |  |  |  | K.VLQAMELMTEKR.I + 2 Oxidation (M5,M8) | 740.8734 | 2+ | 1479.7426 | -6-7 |  |  |  |  |  |  |  |  |
|  |  |  |  |  |  |  |  |  | K.NMIGMVSIGDVVR.A + 2 Oxidation (M2, M5) | 711.8580 | 2+ | 1421.7007 | 0.5 |  |  |  |  |  |  |  |  |
| 19 | Chaperone | *Agave tequilana* | gi\|99033691 | 19.0/6.20 | 18.2/5.84 | 184 | 4 | -- | R.SDVGGALTEASTLANTR.I | 831.9163 | 2+ | 1661.8220 | -2.0 | As | gi\|378346866 | 1 | 2E-67 | 5 | 0.23 |  | |
|  |  |  |  |  |  |  |  |  | K.EEVKVEVEEGR.V | 651.8334 | 2+ | 1301.6463 | 5.0 |  |  |  |  |  |  |  |  |
|  |  |  |  |  |  |  |  |  | R.LPENAKVEEVK.A | 628.3487 | 2+ | 1254.6819 | 1.0 |  |  |  |  |  |  |  |  |
|  |  |  |  |  |  |  |  |  | K.ASMENGVLTVMVPK.V | 738.3836 | 2+ | 1474.7524 | 0.0 |  |  |  |  |  |  |  |  |
| 20 | 18.1 kDa class I heat shock protein-like | *Musa acuminata subsp. malaccensis* | gi\|695043589 | 20.0/5.97 | 18.6/5.10 | 129 | 2 | 15% | K.EEVKVEIEDGR.F | 651.8336 | 2+ | 1301.6463 | 4.9 | NCBI | --- | -- | --- | 5 | 0.24 |  | |
|  |  |  |  |  |  |  |  |  | R.AAMEDGVLTVTVPK.E + Oxidation (M3) | 723.8777 | 2+ | 1445.7436 | -1.9 |  |  |  |  |  |  |  |  |
| 21 | Glutathione S-transferase DHAR2-like | *Oryza brachyantha* | gi\|573943193 | 25.0/5.79 | 23.6/5.82 | 703 | 13 | -- | K.AAYGHPDAVGDCPFSQR.V | 616.6032 | 3+ | 1846.8057 | -10.0 | As | gi\|378361081 | 2 | 2E-111 | 1 | 0.47 |  | |
|  |  |  |  |  |  |  |  |  | R.VTLTLEEK.K | 466.7666 | 2+ | 931.5226 | -4.0 |  |  |  |  |  |  |  |  |
|  |  |  |  |  |  |  |  |  | K.KIPYEYK.L | 470.7602 | 2+ | 939.5065 | -1.0 |  |  |  |  |  |  |  |  |
|  |  |  |  |  |  |  |  |  | K.IPYEYK.L | 406.7125 | 2+ | 811.4116 | -1.0 |  |  |  |  |  |  |  |  |
|  |  |  |  |  |  |  |  |  | K.LVDLSNKPSWFLEINPEGK.V | 729.3832 | 3+ | 2185.1419 | -6.0 |  |  |  |  |  |  |  |  |
|  |  |  |  |  |  |  |  |  | K.VPVYKDEEGK.W | 582.3009 | 2+ | 1162.5870 | 0.0 |  |  |  |  |  |  |  |  |
|  |  |  |  |  |  |  |  |  | K.WIADSDVITK.I | 574.3043 | 2+ | 1146.5921 | 2.0 |  |  |  |  |  |  |  |  |
|  |  |  |  |  |  |  |  |  | K.IIEEKYPEPSLVTPPEYSSVGSK.I | 850.4369 | 3+ | 2548.2948 | -2.0 |  |  |  |  |  |  |  |  |
|  |  |  |  |  |  |  |  |  | K.SKDPNDGSEQALLAELQALDDHLK.A | 652.5740 | 4+ | 2606.2823 | -6.0 |  |  |  |  |  |  |  |  |
|  |  |  |  |  |  |  |  |  | K.DPNDGSEQALLAELQALDDHLK.A | 798.0580 | 3+ | 2391.1554 | -1.0 |  |  |  |  |  |  |  |  |
|  |  |  |  |  |  |  |  |  | K.AHGPFINGENISAADLSLAPK.L | 708.0384 | 3+ | 2121.0854 | 4.0 |  |  |  |  |  |  |  |  |
|  |  |  |  |  |  |  |  |  | K.VPESLPNVNAYMK.L + Oxidation (M12) | 739.3697 | 2+ | 1476.7282 | -2.0 |  |  |  |  |  |  |  |  |
|  |  |  |  |  |  |  |  |  | R.DHVIAGWEPK.V | 576.2989 | 2+ | 1150.5771 | 5.0 |  |  |  |  |  |  |  |  |
| 22 | Glutathione S-transferase DHAR2-like | *Oryza brachyantha* | gi\|573943193 | 24.0/5.29 | 23.6/5.82 | 653 | 9 | -- | K.LVDLSNKPSWFLEINPEGK.V | 729.3870 | 3+ | 2185.1419 | -1.0 | As | gi\|378361081 | 2 | 2E-111 | 1 | 0.67 |  | |
|  |  |  |  |  |  |  |  |  | K.WIADSDVITK.I | 574.3052 | 2+ | 1146.5921 | 3.0 |  |  |  |  |  |  |  |  |
|  |  |  |  |  |  |  |  |  | K.IIEEKYPEPSLVTPPEYSSVGSK.I | 850.4362 | 3+ | 2548.2948 | -3.0 |  |  |  |  |  |  |  |  |
|  |  |  |  |  |  |  |  |  | K.YPEPSLVTPPEYSSVGSK.I | 968.9791 | 2+ | 1935.9466 | -2.0 |  |  |  |  |  |  |  |  |
|  |  |  |  |  |  |  |  |  | K.SKDPNDGSEQALLAELQALDDHLK.A | 652.5760 | 4+ | 2606.2823 | -3.0 |  |  |  |  |  |  |  |  |
|  |  |  |  |  |  |  |  |  | K.AHGPFINGENISAADLSLAPK.L | 708.0348 | 3+ | 2121.0854 | -1.0 |  |  |  |  |  |  |  |  |
|  |  |  |  |  |  |  |  |  | K.LYHLDIALDHFK.G | 742.9050 | 2+ | 1483.7823 | 9.0 |  |  |  |  |  |  |  |  |
|  |  |  |  |  |  |  |  |  | K.VPESLPNVNAYMK.L + Oxidation (M12) | 739.3719 | 2+ | 1476.7282 | 1.0 |  |  |  |  |  |  |  |  |
|  |  |  |  |  |  |  |  |  | R.DHVIAGWEPK.V | 576.2966 | 2+ | 1150.5771 | 1.0 |  |  |  |  |  |  |  |  |
| 23 | Glutathione S-transferase DHAR2-like | *Oryza brachyantha* | gi\|573943193 | 24.0/5.62 | 23.6/5.82 | 1597 | 8 | -- | K.LVDLSNKPSWFLEINPEGK.V | 729.3913 | 3+ | 2185.1419 | 5.0 | As | gi\|378361081 | 2 | 2E-111 | 1 | 0.69 |  | |
|  |  |  |  |  |  |  |  |  | K.VPVYKDEEGK.W | 582.2977 | 2+ | 1162.5870 | -5.0 |  |  |  |  |  |  |  |  |
|  |  |  |  |  |  |  |  |  | K.WIADSDVITK.I | 574.3021 | 2+ | 1146.5921 | -2.0 |  |  |  |  |  |  |  |  |
|  |  |  |  |  |  |  |  |  | K.SKDPNDGSEQALLAELQALDDHLK.A | 652.5775 | 4+ | 2606.2823 | -1.0 |  |  |  |  |  |  |  |  |
|  |  |  |  |  |  |  |  |  | K.DPNDGSEQALLAELQALDDHLK.A | 798.0595 | 3+ | 2391.1554 | 1.0 |  |  |  |  |  |  |  |  |
|  |  |  |  |  |  |  |  |  | K.AHGPFINGENISAADLSLAPK.L | 708.0313 | 3+ | 2121.0854 | -6.0 |  |  |  |  |  |  |  |  |
|  |  |  |  |  |  |  |  |  | K.VPESLPNVNAYMK.L + Oxidation (M12) | 739.3709 | 2+ | 1476.7282 | -1.0 |  |  |  |  |  |  |  |  |
|  |  |  |  |  |  |  |  |  | R.DHVIAGWEPK.V | 576.2947 | 2+ | 1150.5771 | -2.0 |  |  |  |  |  |  |  |  |
| 24 | Glutathione S-transferase DHAR2-like | *Oryza brachyantha* | gi\|573943193 | 24.0/5.46 | 23.6/5.82 | 1024 | 8 | -- | K.KIPYEYK.L | 470.7580 | 2+ | 939.5065 | -5.0 | As | gi\|378361081 | 2 | 2E-111 | 1 | 0.51 |  | |
|  |  |  |  |  |  |  |  |  | K.LVDLSNKPSWFLEINPEGK.V | 729.3888 | 3+ | 2185.1419 | 1.0 |  |  |  |  |  |  |  |  |
|  |  |  |  |  |  |  |  |  | K.WIADSDVITK.I | 574.3010 | 2+ | 1146.5921 | -4.0 |  |  |  |  |  |  |  |  |
|  |  |  |  |  |  |  |  |  | K.IIEEKYPEPSLVTPPEYSSVGSK.I | 850.4383 | 3+ | 2548.2948 | -1.0 |  |  |  |  |  |  |  |  |
|  |  |  |  |  |  |  |  |  | K.YPEPSLVTPPEYSSVGSK.I | 968.9836 | 2+ | 1935.9466 | 3.0 |  |  |  |  |  |  |  |  |
|  |  |  |  |  |  |  |  |  | K.SKDPNDGSEQALLAELQALDDHLK.A | 652.5718 | 4+ | 2606.2823 | -9.0 |  |  |  |  |  |  |  |  |
|  |  |  |  |  |  |  |  |  | K.AHGPFINGENISAADLSLAPK.L | 708.0348 | 3+ | 2121.0854 | -1.0 |  |  |  |  |  |  |  |  |
|  |  |  |  |  |  |  |  |  | K.VPESLPNVNAYMK.L + Oxidation (M12) | 739.3709 | 2+ | 1476.7282 | -1.0 |  |  |  |  |  |  |  |  |
| 25 | Glutathione S-transferase | *Allium cepa* | gi\|144226181 | 26.0/5.98 | 23.7/5.78 | 107 | 2 | -- | K.KPEFLQINPFGQVPALEDGDIK.L | 819.0984 | 3+ | 2454.2795 | -2.0 | As | gi\|378352835 | 2 | 2E-140 | 1 | 0.54 |  | |
|  |  |  |  |  |  |  |  |  | K.YKESGTDLLPAK.T | 661.3553 | 2+ | 1320.6925 | 3.0 |  |  |  |  |  |  |  |  |
| 26 | Glutathione S-transferase T1-like | *Musa acuminata subsp. malaccensis* | gi\|695077436 | 29.0/6.54 | 28.1/9.10 | 536 | 9 | -- | K.FNNIDFEEVK.I | 627.8079 | 2+ | 1253.5928 | 7.0 | As | gi\|378361165 | 1 | 4E-88 | 1 | 0.64 |  | |
|  |  |  |  |  |  |  |  |  | K.FNNIDFEEVKIELFK.G | 628.9970 | 3+ | 1883.9669 | 1.0 |  |  |  |  |  |  |  |  |
|  |  |  |  |  |  |  |  |  | K.VAEHWYPTDLK.K | 679.8446 | 2+ | 1357.6666 | 6.0 |  |  |  |  |  |  |  |  |
|  |  |  |  |  |  |  |  |  | R.ARVDTILDWHHTHTR.L | 619.9857 | 3+ | 1856.9394 | -2.0 |  |  |  |  |  |  |  |  |
|  |  |  |  |  |  |  |  |  | R.LGSAPFVVHTVLGPILGLPLNPEAAK.E | 653.3795 | 4+ | 2609.4945 | -2.0 |  |  |  |  |  |  |  |  |
|  |  |  |  |  |  |  |  |  | K.ILESSLSSIESLWLK.G | 850.9757 | 2+ | 1703.9345 | 1.0 |  |  |  |  |  |  |  |  |
|  |  |  |  |  |  |  |  |  | K.KILQLIENVK.A | 599.3792 | 2+ | 1196.7492 | -4.0 |  |  |  |  |  |  |  |  |
|  |  |  |  |  |  |  |  |  | K.ILQLIENVK.A | 535.3364 | 2+ | 1068.6543 | 4.0 |  |  |  |  |  |  |  |  |
|  |  |  |  |  |  |  |  |  | K.AFTNPHFEEVHQTLYR.T | 663.6585 | 3+ | 1987.9541 | 0.0 |  |  |  |  |  |  |  |  |
| 27 | Nascent polypeptide-associated complex subunit | *Musa acuminata subsp. malaccensis* | gi\|695027681 | 27.0/4.53 | 21.8/4.39 | 118 | 3 | -- | K.NILFVISKPDVFK.S | 760.4498 | 2+ | 1518.8810 | 3.0 | As | gi\|378351378 | 2 | 2E-79 | 4 | 4.20 |  | |
|  |  |  |  |  |  |  |  |  | K.SPNSDTYVIFGEAK.I | 764.3718 | 2+ | 1526.7253 | 2.0 |  |  |  |  |  |  |  |  |
|  |  |  |  |  |  |  |  |  | K.DIELVMTQAGVSRPK.A | 548.6288 | 3+ | 1642.8712 | -4.0 |  |  |  |  |  |  |  |  |
| 28 | Not identified | *---* | --- | 29.0/6.24 | --- | --- | --- | --- | --- | --- | --- | --- | --- | --- | --- | --- | --- | --- | 0.60 |  | |
| 29 | Not identified | *---* | --- | 30.0/6.36 | --- | --- | --- | --- | --- | --- | --- | --- | --- | --- | --- | --- | --- | --- | 0.52 |  | |
| 30 | Not identified | *---* | --- | 34.0/6.66 | --- | --- | --- | --- | --- | --- | --- | --- | --- | --- | --- | --- | --- | --- | 0.62 |  | |
| 31 | Coat protein | *Garlic common latent virus* | gi\|99867305 | 38.0/5.93 | 35.3/6.68 | 416 | 6 | -- | R.QDAAIDSEEPADVQETSVNDVDLR.Q | 872.7357 | 3+ | 2615.1835 | 1.0 | As | gi\|378346192 | 2 | 3E-175 | 9 | 0.25 |  | |
|  |  |  |  |  |  |  |  |  | K.AESLAVATAEDLAAITAK.F | 872.9771 | 2+ | 1743.9254 | 8.0 |  |  |  |  |  |  |  |  |
|  |  |  |  |  |  |  |  |  | K.FEQLGVPTER.L | 588.3081 | 2+ | 1174.5982 | 3.0 |  |  |  |  |  |  |  |  |
|  |  |  |  |  |  |  |  |  | R.YCADTSSSYVADPK.G | 782.3314 | 2+ | 1562.6559 | -5.0 |  |  |  |  |  |  |  |  |
|  |  |  |  |  |  |  |  |  | K.GTFEYPGGAITR.D | 634.8195 | 2+ | 1267.6197 | 4.0 |  |  |  |  |  |  |  |  |
|  |  |  |  |  |  |  |  |  | R.AFAPVVWNEMLIAK.R + Oxidation (M10) | 802.9320 | 2+ | 1603.8432 | 4.0 |  |  |  |  |  |  |  |  |
| 32 | Coat protein | *Garlic common latent virus* | gi\|99867305 | 40.0/6.56 | 35.3/6.68 | 845 | 7 | -- | K.KFQADNMTAGEIK.N | 726.8598 | 2+ | 1451.7078 | -2.0 | As | gi\|378346192 | 2 | 3E-175 | 9 | 0.13 |  | |
|  |  |  |  |  |  |  |  |  | K.NGGFETGRPK.L | 531.7681 | 2+ | 1061.5254 | -4.0 |  |  |  |  |  |  |  |  |
|  |  |  |  |  |  |  |  |  | R.GDTANVFTRPSMDALIALDFK.A | 761.3901 | 3+ | 2281.1413 | 3.0 |  |  |  |  |  |  |  |  |
|  |  |  |  |  |  |  |  |  | K.AESLAVATAEDLAAITAK.F | 872.9706 | 2+ | 1743.9254 | 1.0 |  |  |  |  |  |  |  |  |
|  |  |  |  |  |  |  |  |  | K.FEQLGVPTER.L | 588.3090 | 2+ | 1174.5982 | 4.0 |  |  |  |  |  |  |  |  |
|  |  |  |  |  |  |  |  |  | K.GTFEYPGGAITR.D | 634.8184 | 2+ | 1267.6197 | 2.0 |  |  |  |  |  |  |  |  |
|  |  |  |  |  |  |  |  |  | R.AFAPVVWNEMLIAK.R + Oxidation (M10) | 802.9368 | 2+ | 1603.8432 | 10.0 |  |  |  |  |  |  |  |  |
| (33) | Adenosine kinase 2-like | *Phoenix dactylifera* | gi\|672135802 | 40.0/5.58 | 37.5/5.18 | 171 | 3 | -- | K.LNDAILAEEK.H | 558.3011 | 2+ | 1114.5870 | 1.0 | As | gi\|378352025 | 2 | 5E-95 | 8 | 2.48 |  | |
|  |  |  |  |  |  |  |  |  | K.YSVEYTAGGATQNSIR.V | 858.9125 | 2+ | 1715.8115 | -1.0 |  |  |  |  |  |  |  |  |
|  |  |  |  |  |  |  |  |  | R.SLIANLSAANCYK.V | 712.8646 | 2+ | 1423.7129 | 1.0 |  |  |  |  |  |  |  |  |
| (33) | Coat protein | *Garlic common latent virus* | gi\|99867305 | 40.0/5.58 | 35.3/6.68 | 125 | 2 | -- | R.GDTANVFTRPSMDALIALDFK.A | 761.3907 | 3+ | 2281.1413 | 4.0 | As | gi\|378346192 | 2 | 3E-175 | 9 |  |  | |
|  |  |  |  |  |  |  |  |  | K.AESLAVATAEDLAAITAK.F | 872.9738 | 2+ | 1743.9254 | 4.0 |  |  |  |  |  |  |  |  |
| (33) | Serine/arginine-rich splicing factor SR34A-like isoform X2 | *Phoenix dactylifera* | gi\|672175858 | 40.0/5.58 | 30.1/10.35 | 108 | 2 | -- | R.ESEIEDIFYK.Y | 636.8051 | 2+ | 1271.5920 | 2.9 | As | gi\|389060678 | 5 | 9E-84 | 3 |  |  | |
|  |  |  |  |  |  |  |  |  | R.TVYVGNLPLDVR.E | 673.3838 | 2+ | 1344.7401 | 9.6 |  |  |  |  |  |  |  |  |
| 34 | Annexin D2-like | *Phoenix dactylifera* | gi\|672117887 | 43.0/6.64 | 35.8/8.21 | 411 | 7 | -- | R.QAYSDTFGEDLLK.A | 743.8587 | 2+ | 1485.6987 | 3.0 | As | gi\|378353564 | 1 | 3E-119 | 4 | 0.53 |  | |
|  |  |  |  |  |  |  |  |  | R.VVLLWTLDPAER.D | 706.4039 | 2+ | 1410.7871 | 4.0 |  |  |  |  |  |  |  |  |
|  |  |  |  |  |  |  |  |  | R.SLEEDVAAHTK.D | 600.3027 | 2+ | 1198.5830 | 7.0 |  |  |  |  |  |  |  |  |
|  |  |  |  |  |  |  |  |  | R.KLLVPLVSSLR.Y | 612.9005 | 2+ | 1223.7965 | -8.0 |  |  |  |  |  |  |  |  |
|  |  |  |  |  |  |  |  |  | R.YDGPEVNISLAK.S | 653.3382 | 2+ | 1304.6612 | 0.0 |  |  |  |  |  |  |  |  |
|  |  |  |  |  |  |  |  |  | R.SKAQLFATFNEYNNDIGHPINK.D | 841.0857 | 3+ | 2520.2397 | -2.0 |  |  |  |  |  |  |  |  |
|  |  |  |  |  |  |  |  |  | K.AQLFATFNEYNNDIGHPINK.D | 769.3758 | 3+ | 2305.1127 | -3.0 |  |  |  |  |  |  |  |  |
| 35 | Malate dehydrogenase | *Musa acuminata subsp. malaccensis* | gi\|695022626 | 43.0/6.30 | 35.5/6.00 | 398 | 8 | -- | R.GIMLGPDQPVILHMLDIPPAAEALNGVK.M | 970.5259 | 3+ | 2908.5554 | 0.0 | As | gi\|378354785 | 1 | 8E-158 | 7 | 1.74 |  | |
|  |  |  |  |  |  |  |  |  | K.MELVDAAFPLLK.G + Oxidation (M1) | 681.8696 | 2+ | 1361.7265 | -1.0 |  |  |  |  |  |  |  |  |
|  |  |  |  |  |  |  |  |  | K.VLVVANPANTNALILK.E | 825.5071 | 2+ | 1648.9876 | 7.0 |  |  |  |  |  |  |  |  |
|  |  |  |  |  |  |  |  |  | K.EFAPSIPEK.N | 509.2667 | 2+ | 1016.5178 | 1.0 |  |  |  |  |  |  |  |  |
|  |  |  |  |  |  |  |  |  | K.VQVSDVK.N | 387.7206 | 2+ | 773.4283 | -2.0 |  |  |  |  |  |  |  |  |
|  |  |  |  |  |  |  |  |  | R.ELVGDDDWLR.G | 609.2930 | 2+ | 1216.5724 | -1.0 |  |  |  |  |  |  |  |  |
|  |  |  |  |  |  |  |  |  | R.ELVGDDDWLRGEFITTVQQR.G | 793.0664 | 3+ | 2376.1710 | 3.0 |  |  |  |  |  |  |  |  |
|  |  |  |  |  |  |  |  |  | R.GEFITTVQQR.G | 589.8138 | 2+ | 1177.6091 | 3.0 |  |  |  |  |  |  |  |  |
| 36 | Heterogeneous nuclear ribonucleoprotein 1-like | *Phoenix dactylifera* | gi\|672123610 | 44.0/6.11 | 47.0/8.36 | 197 | 6 | -- | K.LFIGGISWETTEENLK.G | 918.9739 | 2+ | 1835.9305 | 1.0 | As | gi\|378346245 | 3 | 8E-97 | 3 | 3.48 |  | |
|  |  |  |  |  |  |  |  |  | K.YGEVLQTVIMR.D + Oxidation (M10) | 662.8530 | 2+ | 1323.6857 | 4.0 |  |  |  |  |  |  |  |  |
|  |  |  |  |  |  |  |  |  | R.GFGFVVFADPSILDR.V | 820.4295 | 2+ | 1638.8406 | 2.0 |  |  |  |  |  |  |  |  |
|  |  |  |  |  |  |  |  |  | R.VLEDTHTIDGR.T | 628.3209 | 2+ | 1254.6204 | 5.0 |  |  |  |  |  |  |  |  |
|  |  |  |  |  |  |  |  |  | K.IFVGGLPASLSEDEFR.Q | 868.9498 | 2+ | 1735.8781 | 4.0 |  |  |  |  |  |  |  |  |
|  |  |  |  |  |  |  |  |  | R.GFGFISFDEEEAVDR.V | 859.3927 | 2+ | 1716.7631 | 4.0 |  |  |  |  |  |  |  |  |
| (37) | Fructose-bisphosphate aldolase cytoplasmic isozyme-like | *Musa acuminata subsp. malaccensis* | gi\|695047866 | 45.0/6.62 | 38.5/7.04 | 243 | 6 | -- | K.GILAADESTGTIGK.R | 666.8550 | 2+ | 1331.6933 | 2.0 | As | gi\|378361177 | 1 | 2E-158 | 7 | 4.01 |  | |
|  |  |  |  |  |  |  |  |  | K.TADGKPFVDVLK.E | 645.3616 | 2+ | 1288.7027 | 5.0 |  |  |  |  |  |  |  |  |
|  |  |  |  |  |  |  |  |  | K.EGGVLPGIK.V | 435.2580 | 2+ | 868.5018 | 0.0 |  |  |  |  |  |  |  |  |
|  |  |  |  |  |  |  |  |  | K.GTIELAGTNGETTTQGHDDLGKR.C | 593.5415 | 4+ | 2370.1412 | -2.0 |  |  |  |  |  |  |  |  |
|  |  |  |  |  |  |  |  |  | K.VLLEGTLLKPNMVTPGSDSAK.V + Oxidation (M12) | 729.3979 | 3+ | 2185.1664 | 2.0 |  |  |  |  |  |  |  |  |
|  |  |  |  |  |  |  |  |  | K.VKPQVIAEYTVR.A | 701.9090 | 2+ | 1401.7980 | 4.0 |  |  |  |  |  |  |  |  |
| (37) | Mitochondrial malate dehydrogenase | *Solanum lycopersicum* | gi\|350536645 | 45.0/6.62 | 36.2/8.87 | 230 | 4 | -- | K.VAILGAAGGIGQPLSLLMK.L + Oxidation (M18) | 913.0319 | 2+ | 1824.0543 | -3.0 | As | gi\|378346341 | 1 | 2E-103 | 7 |  |  | |
|  |  |  |  |  |  |  |  |  | R.DDLFNINAGIVK.N | 659.8572 | 2+ | 1317.6929 | 5.0 |  |  |  |  |  |  |  |  |
|  |  |  |  |  |  |  |  |  | R.KLFGVTTLDVVR.A | 674.4047 | 2+ | 1346.7922 | 2.0 |  |  |  |  |  |  |  |  |
|  |  |  |  |  |  |  |  |  | K.LFGVTTLDVVR.A | 610.3590 | 2+ | 1218.6972 | 5.0 |  |  |  |  |  |  |  |  |
| 38 | S-adenosylmethionine synthase/Methionine adenosyltransferase | *Dendrobium crumenatum* | gi\|75306070 | 49.0/5.91 | 43.2/5.42 | 523 | 10 | -- | K.VLVNIQQQSPDIAQGVHGHFTK.K | 604.8259 | 4+ | 2415.2659 | 4.0 | As | gi\|378345708 | 2 | 2E-131 | 1 | 8.09 |  | |
|  |  |  |  |  |  |  |  |  | K.KPEEIGAGDQGHMFGYATDETPELMPLTH  VLATK.L + Oxidation (M13) | 740.7620 | 5+ | 3698.7596 | 4.0 |  |  |  |  |  |  |  |  |
|  |  |  |  |  |  |  |  |  | K.TQVTVEYR.N | 498.2602 | 2+ | 994.5084 | -3.0 |  |  |  |  |  |  |  |  |
|  |  |  |  |  |  |  |  |  | R.NDQGAMVPIR.V + Oxidation (M6) | 558.7811 | 2+ | 1115.5393 | 7.0 |  |  |  |  |  |  |  |  |
|  |  |  |  |  |  |  |  |  | R.VHTVLISTQHDETVTNDEIAK.D | 784.0673 | 3+ | 2349.1812 | 0.0 |  |  |  |  |  |  |  |  |
|  |  |  |  |  |  |  |  |  | K.DLKEHVIKPVVPAQYLDENTIFHLNPSGR.F | 666.7597 | 5+ | 3328.7568 | 2.0 |  |  |  |  |  |  |  |  |
|  |  |  |  |  |  |  |  |  | K.EHVIKPVVPAQYLDENTIFHLNPSGR.F | 744.1409 | 4+ | 2972.5508 | -5.0 |  |  |  |  |  |  |  |  |
|  |  |  |  |  |  |  |  |  | R.FVIGGPHGDAGLTGR.K | 727.3856 | 2+ | 1452.7474 | 6.0 |  |  |  |  |  |  |  |  |
|  |  |  |  |  |  |  |  |  | K.IIIDTYGGWGAHGGGAFSGK.D | 655.3261 | 3+ | 1962.9588 | -1.0 |  |  |  |  |  |  |  |  |
|  |  |  |  |  |  |  |  |  | K.DPTKVDR.S | 415.7218 | 2+ | 829.4294 | 0.0 |  |  |  |  |  |  |  |  |
| 39 | Plasminogen activator inhibitor 1 RNA-binding protein-like | *Musa acuminata subsp. malaccensis* | gi\|695056475 | 54.0/6.31 | 41.7/7.71 | 120 | 2 | -- | R.RGGYGGNVEGDAGGDTERPPR.R | 706.3294 | 3+ | 2115.9682 | -1.0 | As | gi\|378363727 | 3 | 4E-06 | 3 | 0.48 |  | |
|  |  |  |  |  |  |  |  |  | R.GGYGGNVEGDAGGDTERPPR.R | 654.2980 | 3+ | 1959.8671 | 3.0 |  |  |  |  |  |  |  |  |
| 40 | Not identified | *---* | --- | 56.0/6.53 | --- | --- | --- | -- | --- | --- | --- | --- | --- | --- | --- | --- | --- | --- | 0.75 |  | |
| 41 | Chaperonin CPN60-2, mitocondrial | *Musa acuminata subsp. malaccensis* | gi\|695064399 | 65.0/5.49 | 61.2/5.76 | 174 | 7 | -- | K.IEFDMLGTCK.K | 615.2828 | 2+ | 1228.5468 | 3.5 | As | gi\|389061077 | 6 | 3E-150 | 5 | 2.09 |  | |
|  |  |  |  |  |  |  |  |  | K.IGVQIIQNALK.A | 598.8720 | 2+ | 1195.7289 | 0.5 |  |  |  |  |  |  |  |  |
|  |  |  |  |  |  |  |  |  | K.LLEQDNTDLGYDAAK.G | 833.4041 | 2+ | 1664.7893 | 2.6 |  |  |  |  |  |  |  |  |
|  |  |  |  |  |  |  |  |  | K.DRVTDALNATK.A | 602.3198 | 2+ | 1202.6255 | -0.3 |  |  |  |  |  |  |  |  |
|  |  |  |  |  |  |  |  |  | K.APAYIIASNAGVEGAVVVGK.L | 943.5242 | 2+ | 1885.0309 | 1.5 |  |  |  |  |  |  |  |  |
|  |  |  |  |  |  |  |  |  | K.AAVEEGIVPGGGVALLYASK.E | 951.0211 | 2+ | 1900.0305 | -1.5 |  |  |  |  |  |  |  |  |
|  |  |  |  |  |  |  |  |  | R.SAIDLSTSDYDKEK.L | 786.3762 | 2+ | 1570.7362 | 1.1 |  |  |  |  |  |  |  |  |
| 42 | 2,3-bisphosphoglycerate-independent phosphoglycerate mutase | *Musa acuminata subsp. malaccensis* | gi\|695068785 | 71.0/5.90 | 61.0/5.38 | 196 | 9 | -- | K.ALEYEDFNMFDR.V + Oxidation (M9) | 783.3346 | 2+ | 1564.6504 | 2.7 | As | gi\|389115869 | 5 | 0.0 | 7 | 3.84 |  | |
|  |  |  |  |  |  |  |  |  | R.GWDAQVLGEAPYK.F | 717.3545 | 2+ | 1432.6986 | -2.9 |  |  |  |  |  |  |  |  |
|  |  |  |  |  |  |  |  |  | K.GIDAQIASGGGR.M | 551.2879 | 2+ | 1100.5574 | 3.5 |  |  |  |  |  |  |  |  |
|  |  |  |  |  |  |  |  |  | K.IYEGEGFNYIK.Q | 666.8281 | 2+ | 1331.6398 | 1.6 |  |  |  |  |  |  |  |  |
|  |  |  |  |  |  |  |  |  | R.YENDWTVVKR.G | 655.3300 | 2+ | 1308.6462 | -0.6 |  |  |  |  |  |  |  |  |
|  |  |  |  |  |  |  |  |  | K.KGIDAQIASGGGR.M | 615.3337 | 2+ | 1228.6523 | 0.4 |  |  |  |  |  |  |  |  |
|  |  |  |  |  |  |  |  |  | K.NSDQYLPPFVIVDENGK.A | 967.9771 | 2+ | 1933.9421 | -1.3 |  |  |  |  |  |  |  |  |
|  |  |  |  |  |  |  |  |  | R.LDQLQLLLK.G | 542.3433 | 2+ | 1082.6699 | 2.0 |  |  |  |  |  |  |  |  |
|  |  |  |  |  |  |  |  |  | K.RGWDAQVLGEAPYK.F | 795.4069 | 2+ | 1588.7997 | -0.2 |  |  |  |  |  |  |  |  |
| 43 | Heat shock 70 kDa protein, mitochondrial | *Malus domestica* | gi\|658060122 | 77.0/5.57 | 72.9/5.78 | 245 | 5 | -- | R.QAVTNPANTLFGTK.R | 731.3893 | 2+ | 1460.7623 | 1.2 | As | gi\|389076603 | 3 | 4E-126 | 5 | 2.15 |  | |
|  |  |  |  |  |  |  |  |  | K.AVITVPAYFNDAQR.Q | 782.9077 | 2+ | 1563.8044 | -2.3 |  |  |  |  |  |  |  |  |
|  |  |  |  |  |  |  |  |  | R.IINEPTAAALSYGMNNK.E | 903.9567 | 2+ | 1805.8982 | 0.4 |  |  |  |  |  |  |  |  |
|  |  |  |  |  |  |  |  |  | K.ETAEAYLGK.S | 491.2460 | 2+ | 980.4814 | -4.1 |  |  |  |  |  |  |  |  |
|  |  |  |  |  |  |  |  |  | R.RFDDPQTQK.E | 567.7836 | 2+ | 1133.5465 | 5.5 |  |  |  |  |  |  |  |  |
| 44 | Malate dehydrogenase | *Musa acuminata subsp. malaccensis* | gi\|695022626 | 42.0/5.69 | 35.5/6.00 | 116 | 2 | -- | K.MELVDAAFPLLK.G | 673.8733 | 2+ | 1345.7315 | 0.0 | As | gi\|378354785 | 1 | 8E-158 | 7 | 2.30 |  | |
|  |  |  |  |  |  |  |  |  | R.GEFITTVQQR.G | 589.8113 | 2+ | 1177.6091 | -1.0 |  |  |  |  |  |  |  |  |
| 45 | S-adenosylmethionine synthase/Methionine adenosyltransferase | *Dendrobium crumenatum* | gi\|75306070 | 47.0/5.82 | 43.2/5.42 | 310 | 5 | -- | K.VLVNIQQQSPDIAQGVHGHFTK.K | 604.8286 | 4+ | 2415.2659 | 8.0 | As | gi\|378345708 | 2 | 2E-131 | 1 | 2.62 |  | |
|  |  |  |  |  |  |  |  |  | K.TQVTVEYR.N | 498.2635 | 2+ | 994.5084 | 4.0 |  |  |  |  |  |  |  |  |
|  |  |  |  |  |  |  |  |  | R.NDQGAMVPIR.V + Oxidation (M6) | 558.7813 | 2+ | 1115.5393 | 8.0 |  |  |  |  |  |  |  |  |
|  |  |  |  |  |  |  |  |  | R.FVIGGPHGDAGLTGR.K | 727.3763 | 2+ | 1452.7474 | -6.0 |  |  |  |  |  |  |  |  |
|  |  |  |  |  |  |  |  |  | K.IIIDTYGGWGAHGGGAFSGK.D | 655.3262 | 3+ | 1962.9588 | -1.0 |  |  |  |  |  |  |  |  |
| 46 | 2,3-bisphosphoglycerate-independent phosphoglycerate mutase | *Musa acuminata subsp. malaccensis* | gi\|695068785 | 72.0/5.80 | 61.0/5.38 | 173 | 6 | -- | R.YENDWTVVK.R | 577.2742 | 2+ | 1152.5452 | -9.9 | As | gi\|389115869 | 5 | 0.0 | 7 | 2.26 |  | |
|  |  |  |  |  |  |  |  |  | R.GWDAQVLGEAPYK.F | 717.3597 | 2+ | 1432.6986 | 4.3 |  |  |  |  |  |  |  |  |
|  |  |  |  |  |  |  |  |  | R.LDQLQLLLK.G | 542.3405 | 2+ | 1082.6699 | -3.2 |  |  |  |  |  |  |  |  |
|  |  |  |  |  |  |  |  |  | K.ALEYEDFNMFDR.V +Oxidation (M9) | 783.3342 | 2+ | 1564.6504 | 2.2 |  |  |  |  |  |  |  |  |
|  |  |  |  |  |  |  |  |  | K.IYEGEGFNYIK.Q | 666.8278 | 2+ | 1331.6398 | 1.0 |  |  |  |  |  |  |  |  |
|  |  |  |  |  |  |  |  |  | K.GIDAQIASGGGR.M | 551.2872 | 2+ | 1100.5574 | 2.2 |  |  |  |  |  |  |  |  |
| 47 | Coat protein | *Garlic common latent virus* | gi\|99867305 | 38.0/6.18 | 35.3/6.68 | 374 | 7 | -- | R.QDAAIDSEEPADVQETSVNDVDLR.Q | 872.7354 | 3+ | 2615.1835 | 0.4 | As | gi\|378353389 | 2 | 1E-157 | 9 | 0.22 |  | |
|  |  |  |  |  |  |  |  |  | K.KFQADNMTAGEIK.N + Oxidation (M8) | 734.8594 | 2+ | 1467.7028 | 1.0 |  |  |  |  |  |  |  |  |
|  |  |  |  |  |  |  |  |  | R.GDTANVFTRPSMDALIALDFK.A + Oxidation (M12) | 766.7175 | 3+ | 2297.1362 | -2.4 |  |  |  |  |  |  |  |  |
|  |  |  |  |  |  |  |  |  | K.AESLAVATAEDLAAITAK.F | 582.3153 | 3+ | 1743.9254 | -0.8 |  |  |  |  |  |  |  |  |
|  |  |  |  |  |  |  |  |  | K.FEQLGVPTER.L | 588.3088 | 2+ | 1174.5982 | 4.1 |  |  |  |  |  |  |  |  |
|  |  |  |  |  |  |  |  |  | K.GTFEYPGGAITR.D | 634.8206 | 2+ | 1267.6197 | 5.5 |  |  |  |  |  |  |  |  |
|  |  |  |  |  |  |  |  |  | R.AFAPVVWNEMLIAK.R | 794.9338 | 2+ | 1587.8483 | 3.0 |  |  |  |  |  |  |  |  |
| 48 | Not identified | *---* | --- | 10.0/4.51 | --- | --- | --- | --- | --- | --- | --- | --- | --- | --- | --- | --- | --- | --- | 0.48 |  | |
| 49 | Coat protein | *Garlic common latent virus* | gi\|99867305 | 34.0/6.42 | 35.3/6.68 | 226 | 3 | -- | K.GDASNIFTRPSMDALLVR.N + Oxidation (M12) | 660.3394 | 3+ | 1977.9942 | 1.1 | As | gi\|378350779 | 2 | 5E-146 | 9 | 0.45 |  | |
|  |  |  |  |  |  |  |  |  | K.YAAFDTFDAVTNK.A | 731.8467 | 2+ | 1461.6776 | 0.9 |  |  |  |  |  |  |  |  |
|  |  |  |  |  |  |  |  |  | K.AAIQPLEGLIR.A | 590.8577 | 2+ | 1179.6975 | 2.8 |  |  |  |  |  |  |  |  |
| 50 | Not identified | *---* | --- | 10.0/5.26 | --- | --- | --- | --- | --- | --- | --- | --- | --- | --- | --- | --- | --- | --- | 0.58 |  | |
| 51 | Chain A, Mannose-Specific Agglutinin (Lectin) | *Allium sativum* | gi\|4389040 | 11.0/4.75 | 11.85/5.12 | 63 | 2 | 24% | K.AVLQSDGNFVVYDAEGR.S | 920.4479 | 2+ | 1838.8799 | 0.7 | NCBI | --- | -- | --- | 2 | 0.24 |  | |
|  |  |  |  |  |  |  |  |  | R.SLWASHSVR.G | 521.7761 | 2+ | 1041.5356 | 2.0 |  |  |  |  |  |  |  |  |
| 52 | Not identified | *---* | --- | 11.0/4.70 | --- | --- | --- | --- | --- | --- | --- | --- | --- | --- | --- | --- | --- | --- | 0.37 |  | |
| 53 | Probable protein Pop3 | *Phoenix dactylifera* | gi\|672203364 | 11.0/5.80 | 12.3/5.41 | 112 | 2 | -- | R.GYAALVPLIGPMK.S + Oxidation (M12) | 673.3835 | 2+ | 1344.7476 | 3.6 | As | gi\|389121105 | 1 | 2E-54 | 1 | 1.84 |  | |
|  |  |  |  |  |  |  |  |  | K.VIVLDYKPTVVR.S | 467.9504 | 3+ | 1400.8391 | -6.9 |  |  |  |  |  |  |  |  |
| (54) | Chain A, Mannose-Specific Agglutinin (Lectin) | *Allium sativum* | gi\|4389040 | 11.0/5.16 | 11.85/5.12 | 116 | 2 | 24% | K.AVLQSDGNFVVYDAEGR.S | 920.4465 | 2+ | 1838.8799 | -0.8 | NCBI | --- | -- | --- | 2 | 0.36 |  | |
|  |  |  |  |  |  |  |  |  | R.SLWASHSVR.G | 521.7713 | 2+ | 1041.5356 | -7.2 |  |  |  |  |  |  |  |  |
| (54) | Nuclear transport factor 2-like | *Cucumis melo* | gi\|659108091 | 11.0/5.16 | 13.6/6.00 | 99 | 2 | -- | K.ITGLPFQQC.K | 503.7549 | 2+ | 1005.50 | 0.0 | As | gi\|389062192 | 6 | 4E-73 | 4 |  |  | |
|  |  |  |  |  |  |  |  |  | K.ITGLPFQQCK.H + Carbamidomethylation (C9) | 567.8024 | 2+ | 1133.59 | 0.0 |  |  |  |  |  |  |  |  |
| 55 | PREDICTED: glycine-rich protein 2-like | *Musa acuminata subsp. malaccensis* | gi\|695010611 | 24.0/5.76 | 21.2/6.08 | 49 | 2 | -- | R.SLAEGESVEFAITEGDDGR.T | 991.4545 | 2+ | 1980.8911 | 1.7 | As | gi\|389105368 | 4 | 2E-35 | 3 | 3.39 |  | |
|  |  |  |  |  |  |  |  |  | K.GFGFITPDDGSDDLFVHQSSIK.S | 794.7152 | 3+ | 2381.1174 | 2.7 |  |  |  |  |  |  |  |  |
| 56 | Not identified | *---* | --- | 10.0/4.68 | --- | --- | --- | --- | --- | --- | --- | --- | --- | --- | --- | --- | --- | --- | 0.27 |  | |
| 57 | Coat protein | *Garlic common latent virus* | gi\|99867305 | 32.0/5.85 | 35.3/6.68 | 312 | 6 | -- | K.AVNDVGVMDR.E + Oxidation (M8) | 546.2562 | 2+ | 1090.5077 | -9.1 | As | gi\|378361292 | 3 | 8E-155 | 9 | 0.33 |  | |
|  |  |  |  |  |  |  |  |  | R.EGFEAVLR.R | 460.7435 | 2+ | 919.4763 | -4.2 |  |  |  |  |  |  |  |  |
|  |  |  |  |  |  |  |  |  | K.GDASNIFTRPSMDALLVR.N + Oxidation (M12) | 660.3363 | 3+ | 1977.9942 | -3.6 |  |  |  |  |  |  |  |  |
|  |  |  |  |  |  |  |  |  | R.NYAPESNNLATAEELAK.I | 917.9442 | 2+ | 1833.8744 | -0.3 |  |  |  |  |  |  |  |  |
|  |  |  |  |  |  |  |  |  | R.DMVVAVIK.E + Oxidation (M2) | 445.7573 | 2+ | 889.4943 | 6.5 |  |  |  |  |  |  |  |  |
|  |  |  |  |  |  |  |  |  | K.YAAFDTFDAVTNK.A | 731.8455 | 2+ | 1461.6776 | -0.8 |  |  |  |  |  |  |  |  |
| 58 | Not identified | *---* | --- | 10.0/5.23 | --- | --- | --- | --- | --- | --- | --- | --- | --- | --- | --- | --- | --- | --- | 0.27 |  | |
| 59 | Polyadenylate-binding protein RBP45-like | *Musa acuminata subsp. malaccensis* | gi\|695060758 | 48.0/5.31 | 49.0/5.52 | 164 | 3 | -- | R.AMTEMDGQYCSTR.Q + 2 Oxidation (M2,M5) | 791.3063 | 2+ | 1580.5905 | 4.8 | As | gi\|34459018 | 2 | 5E-112 | 3 | 4.36 |  | |
|  |  |  |  |  |  |  |  |  | R.QVFSAYGEVVNIK.I | 727.3925 | 2+ | 1452.7613 | 6.3 |  |  |  |  |  |  |  |  |
|  |  |  |  |  |  |  |  |  | K.LNGTTLGGQSVR.L | 601.8244 | 2+ | 1201.6415 | -6.0 |  |  |  |  |  |  |  |  |
| 60 | Not identified | *---* | --- | 19.0/5.57 | --- | --- | --- | --- | --- | --- | --- | --- | --- | --- | --- | --- | --- | --- | √ |  | |
| 61 | S-adenosylmethionine synthase | *Malus domestica* | gi\|283837546 | 49.0/5.69 | 24.5/4.79 | 314 | 6 | -- | K.TNMVMVFGEITTK.A + 2 Oxidation (M3,M5) | 751.8677 | 2+ | 1501.7157 | 3.5 | As | gi\|378350834 | 3 | 9E-145 | 1 | √ |  | |
|  |  |  |  |  |  |  |  |  | R.SIGFVSDDVGLDADNCK.V | 906.4102 | 2+ | 1810.8044 | 0.8 |  |  |  |  |  |  |  |  |
|  |  |  |  |  |  |  |  |  | K.VLVNIEQQSPDIAQGVHGHFTK.K | 806.4184 | 3+ | 2416.2499 | -6.9 |  |  |  |  |  |  |  |  |
|  |  |  |  |  |  |  |  |  | K.NGTCPWLRPDGK.T | 700.8415 | 2+ | 1399.6667 | 1.3 |  |  |  |  |  |  |  |  |
|  |  |  |  |  |  |  |  |  | R.NDQGAMVPIR.V + Oxidation (M6) | 558.7813 | 2+ | 1115.5393 | 7.8 |  |  |  |  |  |  |  |  |
|  |  |  |  |  |  |  |  |  | R.VHTVLISTQHDETVTNDEIAK.D | 784.0687 | 3+ | 2349.1812 | 1.3 |  |  |  |  |  |  |  |  |
| (62) | Chloroplast ribulose-1,5-bisphosphate carboxylase/oxygenase small subunit | *Musa acuminata* AAA Group | gi\|76574234 | 13.0/6.24 | 18.2/9.05 | 95 | 3 | -- | R.ETGNSPGYYDGR.Y | 658.2810 | 2+ | 1314.5477 | -0.2 | As | gi\|389054457 | 6 | 3E-54 | 6 | √ |  | |
|  |  |  |  |  |  |  |  |  | K.QIEYLIR.N | 467.7678 | 2+ | 933.5283 | -7.8 |  |  |  |  |  |  |  |  |
|  |  |  |  |  |  |  |  |  | K.KFETLSYLPPLTDEQLLK.Q | 712.3968 | 2+ | 2134.1561 | 5.8 |  |  |  |  |  |  |  |  |
| (62) | Peptidyl-prolyl cis-trans isomerase FKBP12 | *Phoenix dactylifera* | gi\|672181595 | 13.0/6.24 | 12.0/7.75 | 83 | 2 | -- | K.DPGQQPFTFK.I | 582.7874 | 2+ | 1163.5611 | -0.8 | As | gi\|389061427 | 4 | 2E-62 | 5 |  |  | |
|  |  |  |  |  |  |  |  |  | K.GQSVTVHCTGFGK.D + Carbamidomethyl (C8) | 689.3355 | 2+ | 1376.6507 | 4.2 |  |  |  |  |  |  |  |  |
| ^a^ Spot numbers as indicated in Figure 1; spot numbers in parentheses indicate that the same spot matched to distinct proteins. ^b^ Plant species represent the most likely orthologous organisms. ^c^ Accession numbers according to NCBInr databse. When the best matches were against the *Allium sativum* transcriptome database, the most likely orthologous obtained after BLASTX against the NCBInr database are reported. ^d^ Experimental mass (kDa) and p*I* of identified protein spots. ^e^ Theoretical mass (kDa) and p*I* of identified proteins retrieved from NCBInr database or after calculation using the compute p*I*/Mw tool (http://web.expasy.org/compute_pi/). ^f^ Mascot score reported after database search. Individual ion scores >23 (for *Allium sativum* subset of the NCBInr EST database), >39 (for the *Viridiplantae* subset of the NCBInr protein database) are statistically significant (*p* < 0.05). ^g^ Number of peptides matched. ^h^ Sequence coverage. ^i^ Database. As = *Allium sativum* subset of the NCBInr EST database; NCBI = *Viridiplantae* subset of the NCBInr protein database. ^j^ Accession numbers according to *Allium sativum* subset of the NCBInr database. ^k^ Translation frame number. ^l^ E-value obtained when *Allium sativum* EST translated (into six frames) sequences were aligned against the NCBInr protein database using BLASTX. ^m^ Biological process according to Gene Ontology (http://www.geneontology.org/), 1. Cellular response to stress, 2. Carbohydrate binding, 3. Regulation of transcription, 4. Transport of macromolecules, 5. Protein folding, 6. Photosynthesis, 7. Carbohydrate metabolism, 8. Nucleotide metabolism, 9. Miscellaneous. ^n^ Fold change is expressed as the ratio of the vol % between low-temperature conditioned/room temperature conditioned samples, and each value represents the mean value of three biologically independent replicates analyzed by duplicate. For some spots, fold change cannot be accurately calculated because of a complete absence of the spot in room temperature conditioned samples; this is noted by the symbols √, indicating the presence of the spot only in low-temperature conditioned samples. ^o^ Protein spot accumulation changes in *Allium sativum* sprouts proteins conditioned at room (23 °C) or low-temperature (5 °C) for five weeks. Each column represents the mean value of three biologically independent replicates analyzed by duplicate. Error bars indicate ± standard error (SE). | | | | | | | | | | | | | | | | | | | | | |
